# Rapid prototyping of arbitrary 2D and 3D wireframe DNA origami

**DOI:** 10.1093/nar/gkab762

**Published:** 2021-09-11

**Authors:** Hyungmin Jun, Xiao Wang, Molly F Parsons, William P Bricker, Torsten John, Shanshan Li, Steve Jackson, Wah Chiu, Mark Bathe

**Affiliations:** Department of Biological Engineering, Massachusetts Institute of Technology, Cambridge, MA 02139, USA; Division of Mechanical System Engineering, Jeonbuk National University, Jeonju-si, Jellabuk-do 54896, Republic of Korea; Department of Biological Engineering, Massachusetts Institute of Technology, Cambridge, MA 02139, USA; Department of Biological Engineering, Massachusetts Institute of Technology, Cambridge, MA 02139, USA; Department of Biological Engineering, Massachusetts Institute of Technology, Cambridge, MA 02139, USA; Department of Biological Engineering, Massachusetts Institute of Technology, Cambridge, MA 02139, USA; Department of Bioengineering, and James H. Clark Center, Stanford University, Stanford, CA 94305, USA; Department of Biological Engineering, Massachusetts Institute of Technology, Cambridge, MA 02139, USA; Department of Bioengineering, and James H. Clark Center, Stanford University, Stanford, CA 94305, USA; SLAC National Accelerator Laboratory, Stanford University, Menlo Park, CA 94025, USA; Department of Biological Engineering, Massachusetts Institute of Technology, Cambridge, MA 02139, USA

## Abstract

Wireframe DNA origami assemblies can now be programmed automatically from the top-down using simple wireframe target geometries, or meshes, in 2D and 3D, using either rigid, six-helix bundle (6HB) or more compliant, two-helix bundle (DX) edges. While these assemblies have numerous applications in nanoscale materials fabrication due to their nanoscale spatial addressability and high degree of customization, no easy-to-use graphical user interface software yet exists to deploy these algorithmic approaches within a single, standalone interface. Further, top-down sequence design of 3D DX-based objects previously enabled by DAEDALUS was limited to discrete edge lengths and uniform vertex angles, limiting the scope of objects that can be designed. Here, we introduce the open-source software package ATHENA with a graphical user interface that automatically renders single-stranded DNA scaffold routing and staple strand sequences for any target wireframe DNA origami using DX or 6HB edges, including irregular, asymmetric DX-based polyhedra with variable edge lengths and vertices demonstrated experimentally, which significantly expands the set of possible 3D DNA-based assemblies that can be designed. ATHENA also enables external editing of sequences using caDNAno, demonstrated using asymmetric nanoscale positioning of gold nanoparticles, as well as providing atomic-level models for molecular dynamics, coarse-grained dynamics with oxDNA, and other computational chemistry simulation approaches.

## INTRODUCTION

Structural DNA nanotechnology was conceived in Ned Seeman's pioneering work (1) in which he postulated that synthetic DNA could be used to program synthetic materials with prescribed nanometer scale structural features. The use of synthetic oligonucleotides by Seeman resulted in extended, crystalline-like self-assembled DNA-based materials without control over their overall size and extent. Over two decades later, building on Seeman's design rules, Paul Rothemund introduced the concept of DNA origami (2) that uses the long, single-stranded DNA genome from the M13mp18 phage, which he used to template dozens to hundreds of shorter, complementary synthetic DNA strands that self-assembled or ‘folded’ to form a single, discrete DNA product with high yield. While M13mp18 is still the most commonly used scaffold for this purpose, Rothemund's approach applies generally to any scaffold length and sequence, which may be produced enzymatically (3,4) or bacterially (5,6). Soon after Rothemund's invention, Douglas *et al.* (7) applied Rothemund's approach to self-assemble 3D objects based on similar design rules, and also released the widely used graphical user interface (GUI) software caDNAno (8) to assist in the manual design of this class of DNA origami in which DNA duplexes are arranged on parallel honeycomb or square lattices, also termed ‘bricklike’ origami. While caDNAno has proven extremely useful for the manual scaffold routing and semi-automated sequence design of complementary staples used to self-assemble or fold bricklike target shapes (7–10), it has limited utility for the relatively new class of wireframe DNA origami assemblies that render complex 2D and 3D geometries using wireframe ‘meshes’ that may be composed of single- (11–13), double- (3,14–16), or six-helix bundle edges (17–19).

Wireframe DNA origami using double crossover (DX), two-helix bundle (2HB) edges was first realized by Yan *et al.* (14) with the self-assembly of tiles, which was later generalized to 2D and 3D DNA origami by Zhang *et al.* (15). Benson *et al.* (11) later rendered polyhedral geometries in DNA semi-automatically using single duplexes, and in 2016, Veneziano *et al.* (3) demonstrated that 3D wireframe geometries based exclusively on DX edges could be designed fully automatically based on target geometry, using DAEDALUS. However, DAEDALUS was restricted to objects composed of edges with lengths that are multiples of 10.5 bp, and uniform vertex angles. In 2019, Jun *et al.* demonstrated a fully automatic design procedure for complex 2D wireframe DNA origami without any restrictions on edge length or geometric symmetry based on DX edges, called PERDIX (16). Soon thereafter, they applied a similar design principle to generate six-helix bundle (6HB) edge 3D assemblies (TALOS) (17) and 2D assemblies (METIS) (18), where the 6HB edge-based 2D and 3D assemblies showed significantly enhanced mechanical stiffness with respect to their DX-edge counterparts. PERDIX, METIS, and TALOS also enabled considerably broader classes of asymmetric and complex geometries to be rendered due to the introduction of continuous, arbitrary edge lengths and vertices, highlighting the capabilities of wireframe DNA origami to construct complex nanoscale materials facilitated by automatic design procedures, for example as subunit vaccine nanoparticles (20). However, to date DAEDALUS remains incapable of rendering DNA sequences for completely arbitrary 3D polyhedra composed of non-discrete edge-lengths and arbitrary vertex angles. Further, no integrated GUI software akin to caDNAno (8) enables the facile design of DNA sequences needed to fabricate any wireframe origami object based on either DAEDALUS, PERDIX, TALOS or METIS. Adenita (21) is one GUI software package that does not treat 2D or 6HB wireframe assemblies, and relies on the outdated version of DAEDALUS that is limited to objects with discrete edge lengths and regular vertex angles.

To enable the fully automated sequence design of any wireframe 2D or 3D DNA origami assembly with DX- or 6HB-edges, with arbitrary edge-lengths and vertex angles, here we present ATHENA. ATHENA consists of a GUI that integrates these capabilities and also offers fundamental algorithmic advances to enable arbitrary edge lengths and angles for the precise design of arbitrary wireframe objects including asymmetric and irregular geometries using either uniformly 6HB (17,18) or DX-based edges (3,16). In addition to fully automated sequence design, ATHENA produces output files including all-atom structures in Protein Data Bank (PDB) (22) format for molecular visualization using tools such as Visual Molecular Dynamics (VMD) (23) or UCSF Chimera (24), all-atom molecular dynamics simulation, or coarse-grained simulation using tools such as oxDNA (25,26) or mrDNA (27), as well as caDNAno files for editing or modifying sequence designs for DNA origami functionalization or other purposes (28), and complete sequence files for ordering staple oligonucleotide strands required for fabrication via one-pot self-assembly. We validate sequence designs for 6HB pentagonal objects using atomic force microscopy (AFM), transmission electron microscopy (TEM) and coarse-grained oxDNA simulations, and for a DX-based asymmetric octahedron using cryo-electron microscopy (cryo-EM). We additionally illustrate the use of ATHENA in the asymmetric 2D placement of gold nanoparticles (AuNPs) with nanometer-scale resolution.

## MATERIALS AND METHODS

### GUI implementation

ATHENA is an open-source (GNU GPLv3) GUI software application (https://github.com/lcbb/athena) that performs fully automated sequence design of 2D and 3D wireframe DNA origami objects based on DX- and 6HB-based edges and any ssDNA scaffold of interest. ATHENA was implemented in Python using the Qt5 libraries. Back-end software packages PERDIX, DAEDALUS, METIS, and TALOS are embedded as binaries.

### Arbitrary edge-lengths and vertices in DAEDALUS

Automatic DX-based sequence design for any target 3D polyhedral geometry using continuous edge-lengths and arbitrary vertices is enabled by algorithmic advances in DAEDALUS. Continuous edge designs enable DNA-based objects to be rendered using continuous, arbitrary edge-lengths and vertex angles, with a single duplex filling the gap in each vertex. Unpaired scaffold nucleotides are used to span the distance between the 3′ and 5′ end between incoming and outgoing edges, which would otherwise be misaligned due to the native twist of B-form DNA. Briefly, the algorithm can handle arbitrary edge lengths and vertex angles for precise design of 3D wireframe objects of asymmetric and irregular geometries, which will be the focus of future work.

### PDB generation

The PDB generation software in ATHENA utilizes the nucleic acid base-level nodes that are output from the routing procedure, and these nodes include information on the sequence, routing, and position of each nucleic acid base. The first step in the PDB generation is to route the base-level node information into sequential nucleic acid strands appropriate for an all-atom model, which is accomplished by a searching algorithm since each base is mapped to the upstream, downstream, and paired bases in the model. Next, the all-atom model is built base-by-base and strand-by-strand by transforming the coordinates of a reference average B-form nucleic acid base structure onto the node-level positions. The all-atom nucleic acid structures used are from the 3DNA parameter set (29), where the coordinates are based on average B-form DNA structures from Olson *et al.* (30). Several ProDy coordinate transformation functions are utilized during PDB generation (31). Single-stranded nucleic acid regions are not included in the node-level routing, so the unpaired coordinates are interpolated from the nearest upstream and downstream base-pairs using a cubic Bézier function, providing a smooth path from arbitrary base-pair coordinates. Due to this interpolation procedure, steric clashes may occur between DNA base-pairs in the vertices. For successful all-atom MD simulations (32,33), minimization of the affected ssDNA regions is necessary prior to standard MD procedures. Previous all-atom MD simulations (17,18,34) successfully utilized the initial structures which were output from DAEDALUS, TALOS, and PERDIX, respectively.

The standard PDB file format (22) has several longstanding limitations for large atomic structures, including limitations on the number of separate chains or nucleic acid strands (62, case-sensitive alphanumeric), the number of total atoms (99,999), the number of residues or nucleic acid bases (9,999), and the spatial dimensions {−999.999, 9999.999} in Ångstroms. This PDB generation software utilizes workarounds for some of these limitations. The atom numbering scheme above index 99,999 utilizes a hybrid base-36 encoding scheme where the first character is case-sensitive alphabetical and the following four characters are base-36 alphanumeric, in theory allowing for >87 million total atoms. The alphabetical first character allows any parser to recognize the switch from base-10 to hybrid base-36 encoding. The residue numbering scheme above index 9999 similarly allows for >2.4 million total residues using the same hybrid base-36 encoding. For larger atomic structures, in particular with spatial dimensions exceeding the standard PDB limitations, the PDBx/mmCIF file format (35) could be utilized, but this is left for future work. Output PDB files are compatible with CHARMM force fields for DNA (36,37), and conversion to AMBER force fields (38) is possible within AmberTools (39).

### Materials

DNA origami staple strands were purchased in 96-well plate format from Integrated DNA Technologies, Inc. at 25-nmole synthesis scale. The staple strands were purified by standard desalting and calibrated to 200 μM based on full yield. Staple strands were mixed in equal volume from the corresponding wells and used directly for DNA origami folding without further purification. 5′ Thiol Modifier (C6 S−S) modified DNA strand was purchased from Integrated DNA Technologies, Inc. at 100-nmole synthesis scale with standard desalting. Nuclease Free Water was purchased from Integrated DNA Technologies, Inc. The 7,249-nt DNA scaffold (M13mp18) was purchased from Guild BioSciences at a concentration of 100 nM. The 2,520-nt DNA scaffold (phPB84) was produced following a phage-based protocol (6). 10× TAE buffer was purchased from Alfa Aesar. Magnesium acetate tetrahydrate (molecular biology grade) was purchased from MilliporeSigma. 1× TAE buffer with 12.5 mM Mg(OAc)_2_ was prepared with 10× TAE buffer and Magnesium acetate tetrahydrate. 5 nm OligoREADY AuNP Conjugation Kit was purchased from Cytodiagnostics Inc. Pierce DTT (Dithiothreitol) was purchased from ThermoFisher Scientific, and illustra NAP-5 columns were purchased from GE Healthcare Life Sciences.

### Origami self-assembly

All 2D pentagonal DNA origami objects were folded with the same protocol. 5 nM of DNA scaffold (M13mp18) was mixed with 20 molar equiv. corresponding staples strands in 1× TAE buffer with 12.5 mM Mg(OAc)_2_, the final volume of the self-assembly solution was 100 μl. The mixture was annealed in a PCR thermocycler: 95°C for 2 min, 70°C to 45°C at a rate of 0.5°C per 20 min, and 45°C to 20°C at a rate of 0.5°C per 10 min. The annealed solution was validated by 1.5% Agarose gel in 1× TAE buffer with 12.5 mM Mg(OAc)_2_ and 1× SybrSafe. Gels were run at 60 V and subsequently imaged under blue light. The annealed solution was diluted into 500 μl with 1× TAE buffer with 12.5 mM Mg(OAc)_2_, and the extra staple strands were removed with MWCO = 100 kDa spin filter concentration columns. The purified DNA origami solution was adjusted to desired concentrations (5 nM) for AFM and TEM imaging.

The DX-based origami object was folded using 40 nM DNA scaffold (phPB84) and 20 molar equivalents of required staple strands in 1× TAE buffer with 12 mM MgCl_2_. The folding mixture was annealed in a thermocycler: 95°C for 5 min, 80°C down to 76°C at a rate of 0.8°C per min, 75°C down to 30°C at a rate of 0.42°C per min, and finally 29°C down to 25°C at a rate of 0.625°C per min. The annealed solution was analysed in 2.5% agarose gel in 1× TBE buffer with 12 mM MgCl_2_ and 1× SybrSafe, run at 65 V in a cold room and imaged under blue light. Following removal of staples and buffer exchange into 1× TAE with 8 mM MgCl_2_ using Amicon Ultra 0.5 ml spin filter columns with MWCO = 100 kDa, the purified folded solution was screened using AFM and cryo-EM imaging.

### Preparation of DNA–AuNP conjugate modified DNA origami

The 5′ thiol modified DNA strand (50 μM) was reduced by DTT (0.1 M) in 0.15 M sodium phosphate buffer (pH 8.5) for 2 h at room temperature. The reaction solution was then purified with a Nap-5 column to remove small molecules from 5′ thiol-DNA strand. The purified 5′ thiol-DNA strand was adjusted to 25 μM in nuclease-free water based on the OD_260 nm_. One vial of lyophilized OligoREADY™ 5 nm AuNP was resuspended in 740 μl of nuclease-free H_2_O. 160 μl of purified 5′ thiol-DNA strand (25 μM) and 100 μl of 1 M NaCl were added to the AuNP suspension. The mixture was incubated at room temperature for 2 h. Excess DNA strand was subsequently removed from MWCO = 100 kDa spin filter concentration columns, and the DNA–AuNP conjugate was concentrated in the meantime. The concentration of DNA-AuNP conjugate was determined by OD_520 nm_.

The DNA–AuNP conjugate was added to purified DNA origami solution (20 nM) in a ratio of 5:1 (AuNP : sites of modification on origami), and the mixtures were incubated in 1× TAE buffer with 12.5 mM Mg(OAc)_2_ at room temperature overnight.

### AFM and TEM imaging

AFM imaging was performed in ‘ScanAsyst mode in fluid’ (Dimension FastScan, Bruker Corporation) with ScanAsyst-Fluid+ or SNL-10 tips (Bruker Inc.). Two microliters of sample (5 nM) were deposited onto freshly cleaved mica (Ted Pella Inc.), and 0.5 to 1.0 μl of NiCl_2_ at a concentration of 100 mM were added to the samples to fix the origami nanostructures on the mica surface. After waiting for ∼30 s for sample adsorption to mica, 80 μl of 1× TAE/Mg^2+^ buffer was added to the samples, and an extra 40 μl of the same buffer was deposited onto the AFM tip. For TEM imaging, 5 μl of DNA origami solution (5 nM) was deposited onto fresh glow discharged carbon film with copper grids (CF200H-CU; Electron Microscopy Sciences Inc., Hatfeld, PA), and the sample was then allowed to absorb onto the surface for 30 s. After the sample solution was blotted from the grid using Whatman 42 filter paper, the grid was placed on 5 μl of freshly prepared 2% uranyl formate with 25 mM NaOH for 10 s. The remaining stain solution on the grid was blotted away using Whatman 42 filter paper and dried under house vacuum prior to imaging. The sample was imaged on a Tecnai FEI with a Gatan camera.

### Cryo-EM data collection and image processing

Triton X-100 was added to concentrated purified origami samples (∼1.3 μM) for a final concentration of 0.025%. The resulting solution of 3 μl was applied to glow-discharged Quantifoil R2/1 300-mesh copper grids and frozen in liquid ethane using a Vitrobot (ThermoFisher Scientific) with 6 s blot. Grids were then imaged on a Talos Arctica G2 scope (ThermoFisher Scientific) with a Falcon 3EC detector, operated at 200 kV and 73,000x magnification (2.008 Å nominal pixel size), using EPU software (ThermoFisher Scientific). Single-particle image processing and 3D reconstruction for DX-based asymmetric octahedron of 63-edge length were performed using EMAN2 (40). All particles were picked manually by *e2boxer.py* in EMAN2. The initial models generated by ATHENA software were low-pass filtered to 60 Å to avoid model bias. The following steps were performed as previously described (17). A total of 3,148 particles were used for final refinement of the DX-based asymmetric octahedron of 63-bp edge length.

### Coarse-grained computer simulations using oxDNA

To demonstrate the applicability of ATHENA generated DNA nanostructures to coarse-grained oxDNA simulations, we performed molecular dynamics (MD) simulations using the oxDNA2 model and oxDNA version 2.4 simulation software (41–44). In comparison to full-atomistic MD simulations, oxDNA provides a coarse-grained approximation to study the thermodynamic and mechanical properties of DNA at longer times scales (41,43). We studied the 6HB-based pentagonal objects of varying edge-lengths (42- to 210-bp) as experimentally prepared. The ATHENA output multimodel PDB files were first converted into oxDNA file format using tacoxDNA (45). All DNA nanostructures were simulated at a salt concentration of 1 M [Na^+^], as suggested to represent experimental conditions (43). After an initial energy minimization (2000 steps), the DNA objects were equilibrated for 30.3 μs (10^7^ steps) at 300 K using the Langevin thermostat (diffusion coefficient 2.5). The equilibrated structures were then simulated for 0.303 ms (10^8^ steps, time step: 0.1515 ps) at 300 K using the Anderson-like john thermostat (diffusion coefficient 2.5). The initial velocities were generated from a Maxwellian distribution. The simulations were visualized using oxView and analyzed using oxDNA analysis tools (44,46). Root mean square fluctuations (RMSF) were calculated with reference to the mean structure.

## RESULTS AND DISCUSSION

**Figure 1.**
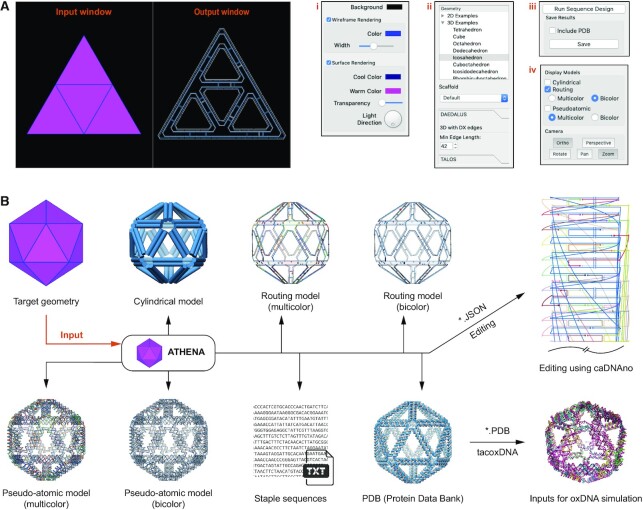
Interface and design outputs of ATHENA. (**A**) Screenshot of graphical user interface that has two windows for rendering the target geometry (input window) and outputs (output window) such as cylindrical, routing, and pseudo-atomic model. Additional four panels are to control options; (i) rendering colour scheme, (ii) target geometry, scaffold sequence, edge length, edge type, (iii) outputs and (iv) camera control. (**B**) Based on the target geometry, DAEDALUS from ATHENA routes a single-stranded scaffold throughout the entire geometry and generates several outputs; cylindrical model, routing model, pseudo-atomic model, text file for staple sequences, JSON for caDNAno, and PDB for molecular dynamics simulations.

In ATHENA, 2D and 3D target geometries are specified using a polygonal surface mesh and, in 3D, each edge of every polygonal surface represents one of the edges of a neighboring surface. These are provided manually or through an ASCII file format that defines the polygonal mesh, such as the Polygon File Format (PLY), STereoLithography (STL), or Virtual Reality Modeling Language (WRL) using any number of CAD programs. ATHENA then provides a fully automated sequence design of 2D or 3D wireframe scaffold DNA origami objects based uniformly either on rigid 6HB or more compliant 2HB edges (Figure 1 and Supplementary Note 1). A PLY file (Supplementary Note 2) is used as input to ATHENA because of its simplicity and broad use within CAD-based designs. Conversion from STL to PLY filetypes may be performed using open-source tools and online convertors. Because scaffold routing and staple design are based on PLY files, it is essential that every vertex listed in the file pertains to at least one face, since otherwise there is no way of routing the ssDNA scaffold through the entire target object (17). Once input is provided, ATHENA offers the ability to visualize the target 2D or 3D wireframe object using surface shading and/or wireframe edges, in default colors that may be altered using custom options (Figure 1A; i). Zooming, rotation, and translation may each be selected as standard mouse options, as well as perspective versus orthographic views (Figure 1A; iv). ATHENA also provides 37 2D and 55 3D pre-defined target geometries (Figure 1A; ii and Supplementary Notes 3 and 4).

ATHENA uses M13mp18 as the default scaffold sequence for required lengths less than or equal to 7,249-nt; a Lambda phage sequence if greater than 7,250-nt and less than or equal to 48,502-nt; and a random sequence if greater than 48,503-nt. User-defined scaffold sequences can also be imported using a text file (Figure 1A; ii). ATHENA has the option to choose the edge type: DNA double-crossover (DX or 2HB) or six-helix bundle (6HB) that consists of every edge of the 2D or 3D wireframe objects (Figure 1A; ii). Then, fully automated scaffold and staple sequence design can be performed using either DX- (3,16) or 6HB-edge (17,18) motifs with either the default, M13 ssDNA scaffold, or a custom scaffold of length and sequence defined by the user (Figure 1A; iii and Supplementary Figure S1). In addition, the minimum edge length is assigned to the shortest edge, which is then used to scale all other edges, specifying from 42-bp (13.9 nm) to 210-bp (71.1 nm) edge-lengths in the design, which may range from 20 to 200 nm for 2D and from 20 to 100 nm for 3D when using the M13mp18 ssDNA scaffold (7,249-nt).

Once the sequence design procedure in ATHENA is completed, a cylindrical representation is displayed overlapping with the target geometry (Figure 1B). In the cylindrical model, each edge of the wireframe structure is rendered using a cylinder (2 nm diameter) that represents a DNA double helix. Strand routing and the helicity of DNA can be displayed using the routing and pseudo-atomic model options (Figure 1A; iv). For the routing model, each strand, including the scaffold and staples, is approximated by a vector representing the direction of the DNA strand (Figure 1B). More detailed output with the double-helical DNA can be displayed in the pseudo-atomic model constructed by spheres and lines representing nucleotides and the backbone of DNA, respectively. For easier identification of the scaffold and individual staples, two-color schemes with multiple colors are built for the routing and pseudo-atomic models. The resulting sequence outputs can also be exported (Figure 1A; iii) with several files; a Comma Separated Values (CSV) spreadsheet containing staple sequences, a PDB all-atom model, and JavaScript Object Notation (JSON) for caDNAno (Figure 1B). The tacoxDNA (45) webserver can be used to convert the PDB file to the appropriate inputs for performing coarse-grained simulations with oxDNA (25,26). The JSON file can be imported into caDNAno (8) for manual base and oligo editing for functionalization, for example, editing sequences, extending strands, deleting and adding nucleotides, and changing the position for crossovers and nicks. ATHENA provides the information on edges of the target structure associated with cross-sections in the caDNAno representation (Supplementary Figure S2).

**Figure 2.**
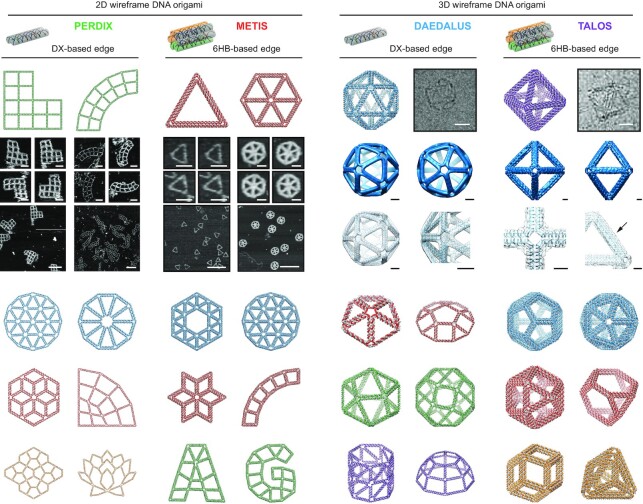
Fully automated sequence design of 2D and 3D DNA wireframe structures with DX- and 6HB-based edges. Scale bars, 50 and 150 nm (zoom-in and zoom-out AFM images, respectively) for PERDIX structure (16), 50 and 200 nm (zoom-in and zoom-out AFM images, respectively) for METIS (18) structure, and 20 nm (cryo-EM image) and 5 nm (cryo-EM reconstruction) for DAEDALUS as extended in this work and TALOS structures (3,17).

Based only on a target geometry, scaffold sequence, and edge type (DX or 6HB), the embedded design algorithms in ATHENA perform automated scaffold routing and staple sequence design, in addition to generating the required staple strands needed to fold the structure experimentally (Figure 2). PERDIX performs fully automated scaffold routing and staple sequence design for any free-form 2D geometry using exclusively DX-based edges, whereas METIS designs any 2D geometry using mechanically stiffer honeycomb or 6HB edges. DAEDALUS solves the scaffold routing and staple design problem fully automatically for any 3D polyhedral surface using solely DX-based edges, whereas TALOS renders any 3D polyhedral surface using mechanically stiffer honeycomb edges, thereby also requiring greater scaffold length for the same particle geometry and size. TALOS additionally offers the ability to utilize every crossover possible between neighbouring 6HB edge duplexes (17), which should offer enhanced mechanical and enzymatic integrity compared with the minimal number of single crossovers utilized between any two edges in previous honeycomb octahedral sequence designs (47).

**Figure 3.**
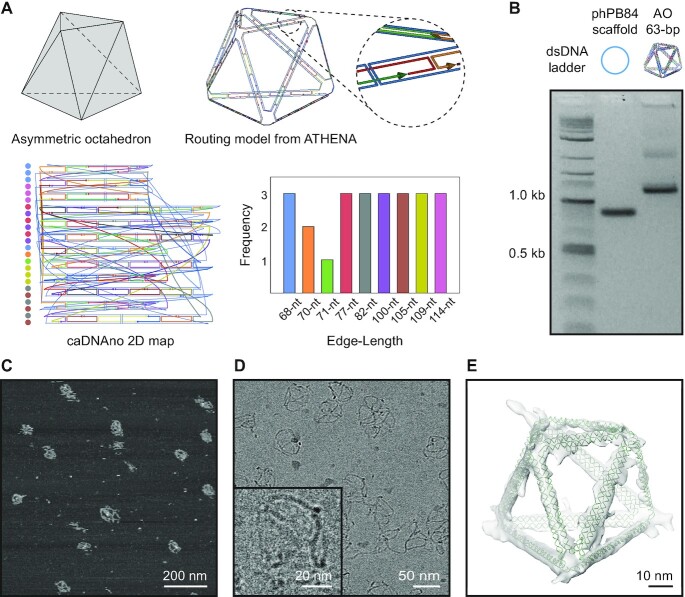
Designing DX-based asymmetric and irregular objects with ATHENA. (**A**) Target geometry and routing model of asymmetric octahedron of 63-bp edge-length. (**B**) Agarose gel electrophoresis for DX-based asymmetric octahedron. (**C**) AFM and (**D**) cryo-EM images of DX-based asymmetric octahedron of 63-bp edge length. (**E**) 3D reconstruction of DX-based asymmetric octahedron of 63-bp edge length using cryo-EM. Solved structure resolution is 3.3 nm.

We tested the ability of ATHENA to generate high-quality wireframe DNA origami structures, which also allows users to further functionalize such structures with other materials conveniently. First, to evaluate the ability of ATHENA to handle arbitrary edge lengths for asymmetric and irregular objects based on DX-edges, we designed an asymmetric octahedron with continuous edge length and variable vertex angles, with 2HB edges. We used a user-input sequence (2,520-nt phPB84, Supplementary Tables S1 and S2) as the scaffold. AFM and cryo-EM confirmed the successful assembly of this structure with high yield (Figure 3 and Supplementary Figure S3), offering the first demonstration of this new sequence design algorithm. Cryo-EM reconstruction of the DX-based asymmetric octahedron of 63-bp edge length also showed that these irregular objects fold as intended, without significant distortion to programmed edges or vertices (Figure 3E).

**Figure 4.**
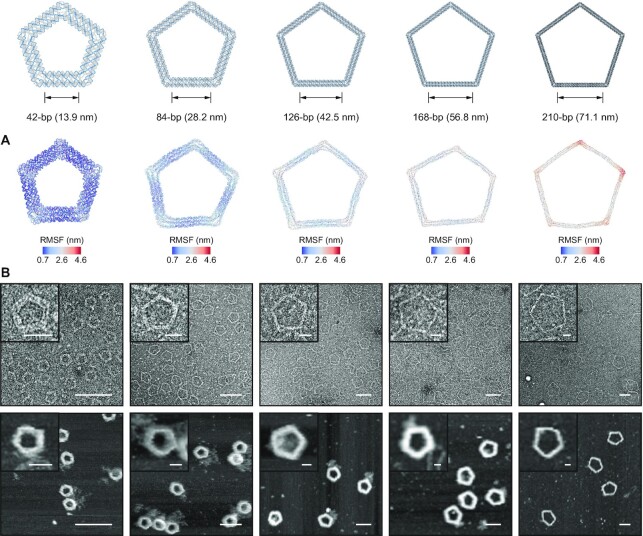
Designing 6HB pentagonal DNA origami objects with variable edge lengths. (**A**) oxDNA simulation results (centroid structures in RMSF coloring), and (**B**) TEM and AFM images for variable edge lengths of 42-, 84-, 126-, 168- and 210-bp DNA pentagonal objects. Scale bars, 25 and 100 nm (zoom-in and zoom-out TEM and AFM images, respectively).

**Figure 5.**
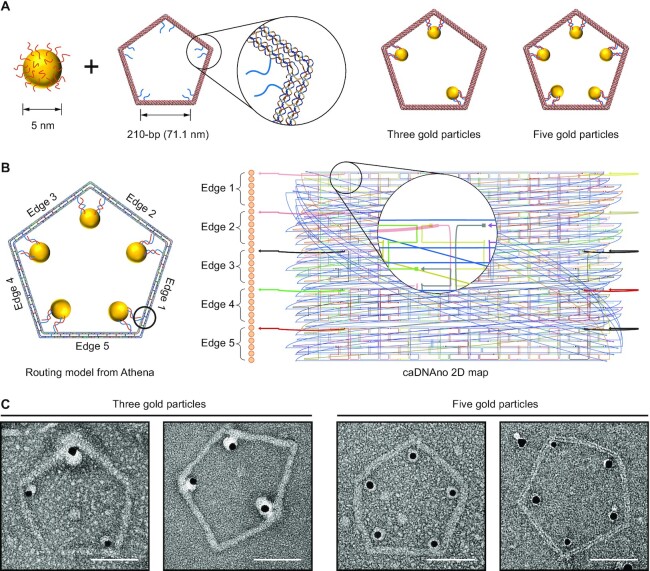
Organizing AuNPs on the 210-bp pentagonal DNA origami. (**A**) Diagrams showing the attachment of NPs at three or five corners. (**B**) Routing model and caDNAno representation for pentagonal DNA origami with AuNPs. (**C**) TEM images with 2D pentagonal DNA origami of organized AuNPs. Scale bars, 50 nm.

Next, we generated the staple strand sequences of five 6HB-based pentagonal objects (Figure 4 and Supplementary Tables S3–S8) with different edge lengths from 42-bp (13.94 nm) to 210-bp (71.06 nm) with ATHENA. TEM and AFM confirmed the successful assembly of target structures as indicated by the accurate vertex angles and the high yield of proper formation of these structures (Supplementary Figures S4–S13). Coarse-grained oxDNA simulations confirmed the high structural stability and rigidity of the structures, particularly those with smaller edge lengths (Figure 4). Users can modify these structures based on the routing and pseudo-atomic model generated by ATHENA, which enables the user to identify the position of a particular modification (nick or overhang position). Each staple strand was labelled with the same color in both the pseudo-atomic model and caDNAno file, for convenience in identifying the corresponding staple strands in the caDNAno file for modifications. To demonstrate the addressability of this well-controlled scaffolding material and editing approach, we modified one of the pentagonal origami structures (210-bp edge length) for AuNP attachment (Figure 5). Following the procedure described in Supplementary Figure S14, we modified staple strands around the vertex of this pentagonal structure for positioning AuNPs. The handles for DNA-AuNP conjugates were placed at either three or all five vertices of the pentagonal structure, and the handles from the adjacent edges were designed to fix one AuNP in the vertex. (Figure 5C and Supplementary Figures S15–S17). TEM images showed that the AuNPs were successfully placed at the prescribed positions in the origami structure, which alternatively could be used to program any number of inorganic or organic molecules, in both 2D and 3D (19).

Taken together, the preceding results demonstrate that ATHENA offers a unified software environment for fully automatic, top-down geometric design of arbitrary wireframe origami designs, including for the first time fully asymmetric 2HB wireframe designs enabled by a new algorithm that accommodates continuous edge lengths and arbitrary vertex angles that cannot be treated using the original version of DAEDALUS (3). Objects folded and modified using ATHENA-generated designs demonstrate the reliability and ease of use of this interface, offering a versatile design tool for a broad array of 2D and 3D wireframe DNA origami objects.

## Supplementary Material

gkab762_Supplemental_File

## Notes

Present address: William P. Bricker, Department of Chemical and Biological Engineering, University of New Mexico, Albuquerque, NM 87131, USA.
