# Supplementary material for: Rapid prototyping of arbitrary 2D and 3D wireframe DNA origami
Source: Nucleic Acids Res. 2021 Sep 11;49(18):10265–74. doi: 10.1093/nar/gkab762 (PMC8501967; doi:10.1093/nar/gkab762)
Supplement: gkab762_Supplemental_File [file gkab762_supplemental_file.pdf]

Rapid Prototyping of Arbitrary 2D and 3D Wireframe DNA Origami

Supplementary Information

Hyungmin Jun[1,4], Xiao Wang[1], Molly Parsons[1], William P. Bricker[1], Torsten John[1], Shanshan Li[2], Steve Jackson[1], Wah Chiu[2,3] and Mark Bathe[1,*]

[1]Department of Biological Engineering, Massachusetts Institute of Technology, Cambridge, MA 02139, USA
[2]Department of Bioengineering, and James H. Clark Center, Stanford University, Stanford, CA 94305, United States
[3]SLAC National Accelerator Laboratory, Stanford University, Menlo Park, CA 94025, United States
[4]Division of Mechanical System Engineering, Jeonbuk National University, Jeonju-si, Jellabuk-do 54896, Republic of Korea
*To whom correspondence should be addressed. Tel: +1 617 324 5685; Email: mark.bathe@mit.edu

Present address: William P. Bricker, Department of Chemical and Biological Engineering, University of New Mexico, Albuquerque, NM 87131, USA

Supplementary Note 1. ATHENA options reference.

Main menu - File

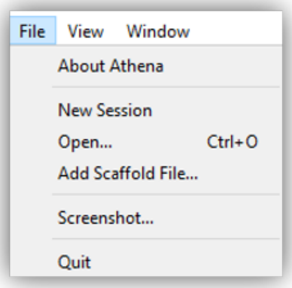

Name	Function
About Athena	Software information
New Session	Make new session
Open	Open custom PLY file
Add Scaffold File	Open the text file for custom scaffold sequences
Screenshot	Captures the working window and export it as an image file
Quit	Quit ATHENA

Main menu - View

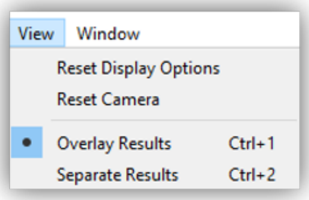

Name	Function
Reset Display	Reset display setting as default
Reset Camera	Reset camera setting as default
Overlay Results	One window; output rendering is overlapped on the target geometry

Separate Results	Two windows; one for the target geometry and another for output rendering

Main menu - Window

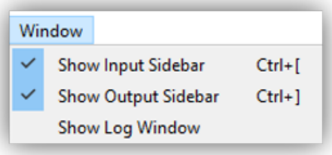

Show Input Sidebar	Show / hide input sidebar
Show Output Sidebar	Show / hide output sidebar
Show Log Window	Log window displays design parameters generated by Athena when it runs

Main menu - Window

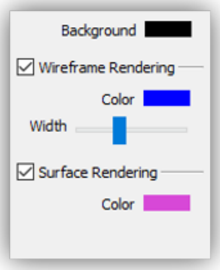

Background	Change the background (black as default)
Wireframe Rendering	Change the color or width of the edge of the target geometry (blue as default)
Surface Rendering	Change the color for the surface of the target geometry (magenta as default)

Display Models

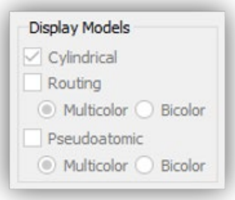

Cylindrical	Cylindrical model representation<br>Each cylinder represents the DNA duplex
Routing	Routing model representation
Pseudoatomic	Pseudoatomic model representation

Camera

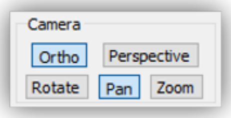

Ortho	Orthographic projection
Perspective	Perspective projection
Rotate	Rotate the object in the view window
Pan	Translate the object in the view window
Zoom	Zoom in/out the object in the view window

Geometry

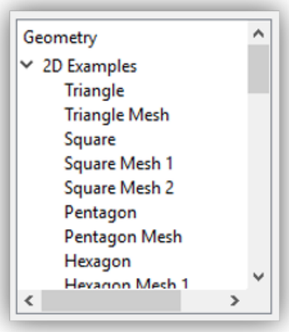

2D Examples	37 pre-defined 2D target geometries
3D Examples	55 pre-defined 3D target geometries
Loaded Files	Display loaded target geometry by user

Scaffold

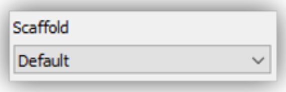

Default	M13mp18 as a default scaffold sequence for required length less than or equal to 7,249-nt, a Lambda phage sequence if greater than 7,250-nt and less or equal to 48,502-nt, and a random sequence if greater than 48,503-nt

PERDIX - DX-based 2D wireframe origami

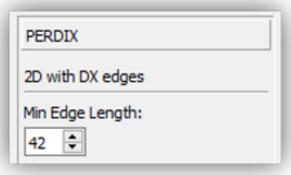

Min Edge Length	The minimum edge length (from 42-bp to 126-bp) is assigned to the shortest edge and the other edges are scaled and rounded appropriately

METIS - 6HB-based 2D wireframe origami

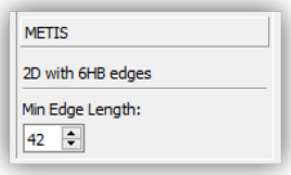

Min Edge Length	The minimum edge length (from 42-bp to 126-bp) is assigned to the shortest edge and the other edges are scaled and rounded appropriately

DAEDALUS - DX-based 3D wireframe origami

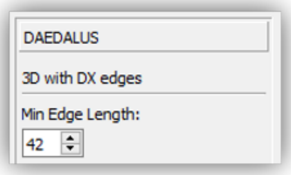

Min Edge Length	The minimum edge length (from 42-bp to 126-bp) is assigned to the shortest edge and the other edges are scaled and rounded appropriately

TALOS - 6HB-based 3D wireframe origami

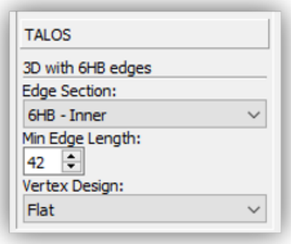

Edge Section	Inner / Middle connections
Min Edge Length	The minimum edge length (from 42-bp to 126-bp) is assigned to the shortest edge and the other edges are scaled and rounded appropriately
Vertex Design	Flat vs mitered vertex design

Run

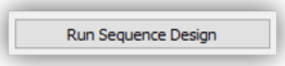

Sequence Design	Run to sequence design

Save Results

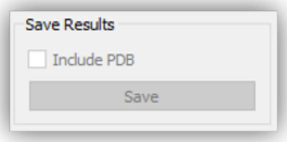

Include PDB	Save outputs with PDB file
Save	Save outputs as files

Supplementary Note 2. Preparing the target geometry design.

ATHENA is compatible with PLY format in ASCII format and users can use general purpose text editors (e.g. Notepad++ and Atom) to create an editable input file (*.PLY) using the following rules.

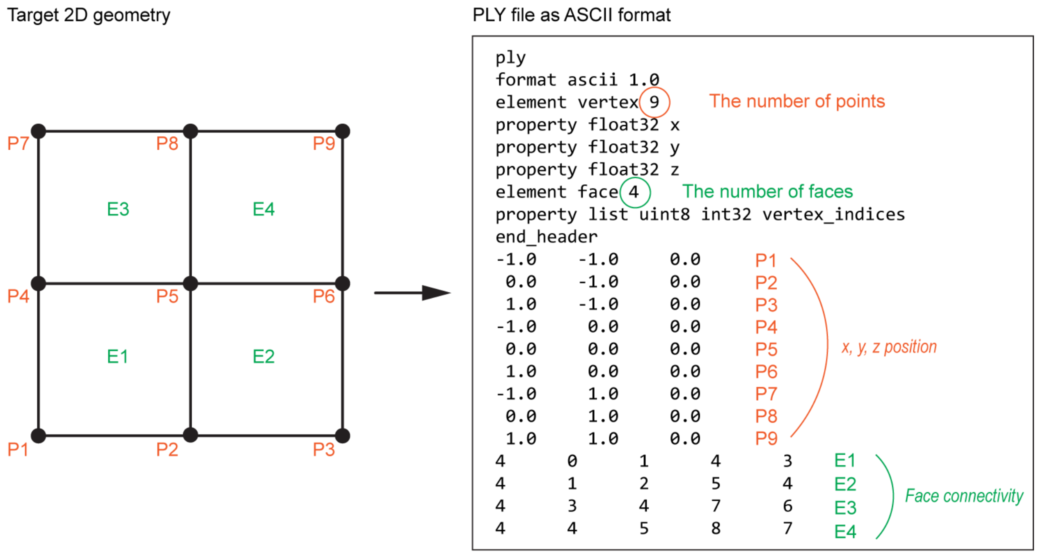

Supplementary Note 3. 37 pre-defined 2D target geometries.

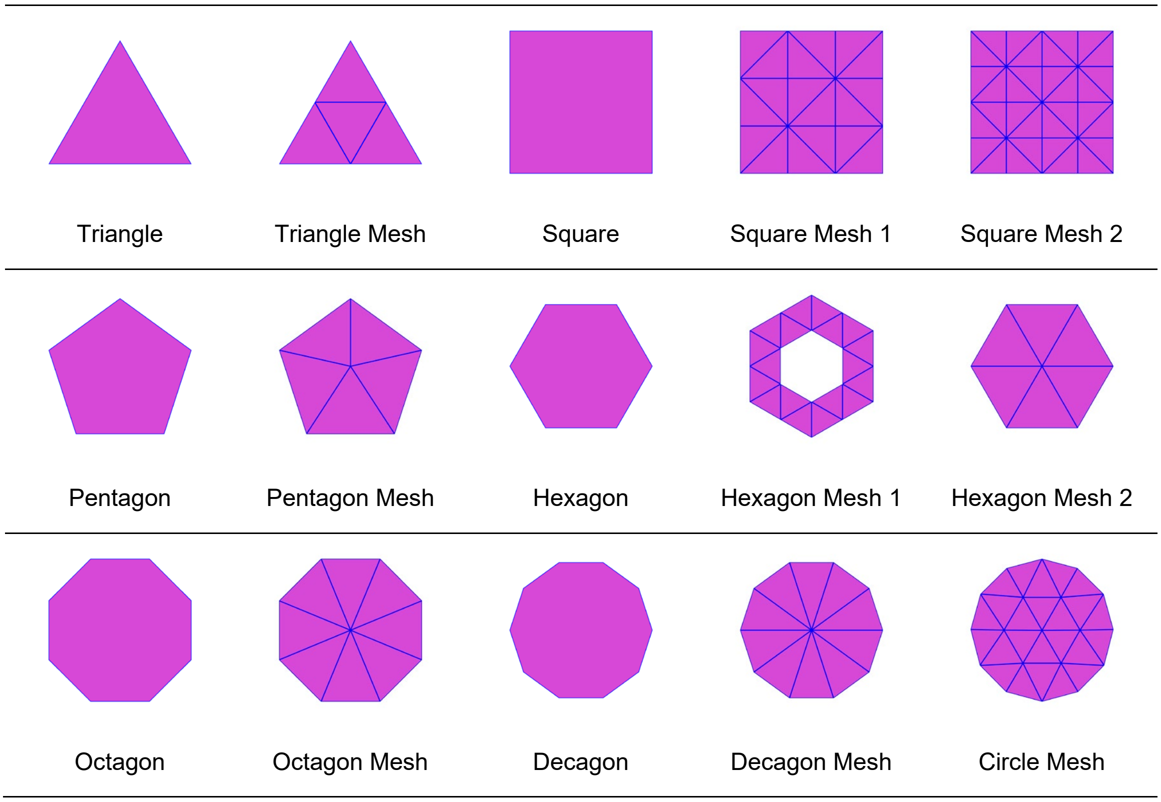

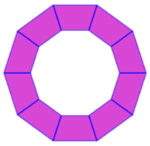
Annulus Mesh 1

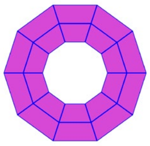
Annulus Mesh 2

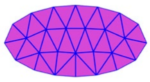
Ellipse Mesh

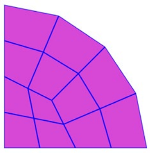
Quarter Circle

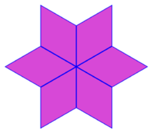
Star Mesh

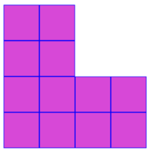
L-shape Mesh

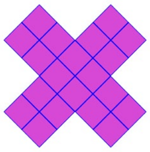
Cross Mesh

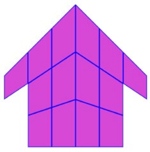
Arrowhead Mesh

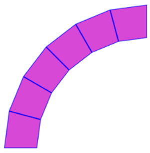
Curve Arm Mesh 1

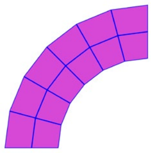
Curve Arm Mesh 2

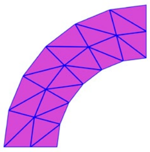
Curve Arm Mesh 3

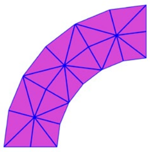
Curve Arm Mesh 4

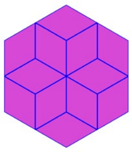
Rhombic Tile

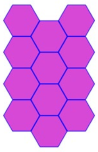
Hexagonal Tile

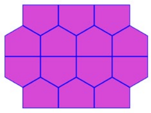
Prismatic Pentagonal tile

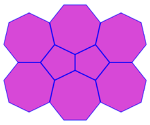
Heptagonal and Pentagonal Tile

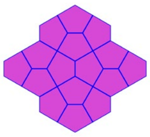
Cairo Pentagonal Tile

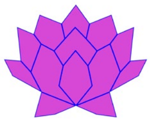
Lotus Mesh

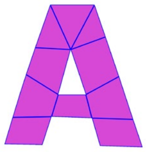
A-Shape Mesh

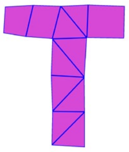
T-Shape Mesh

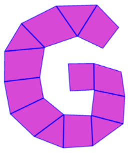
G-Shape Mesh

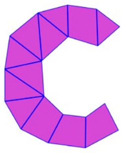
C-Shape Mesh

Supplementary Note 4. 55 pre-defined 3D target geometries.

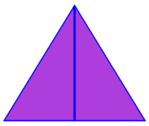

Tetrahedron

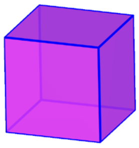

Cube

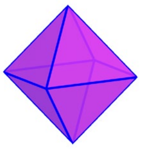

Octahedron

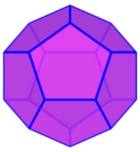

Dodecahedron

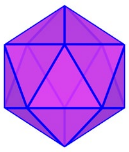

Icosahedron

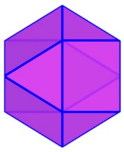

Cuboctahedron

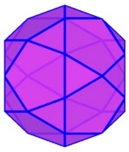

Icosidodecahedron

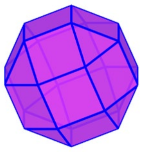

Rhombicuboctahedron

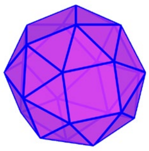

Snub Cube

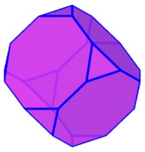

Truncated Cube

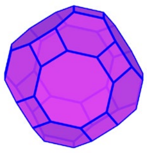

Truncated Cuboctahedron

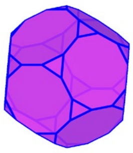

Truncated Dodecahedron

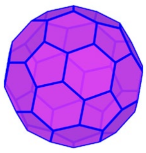

Truncated Icosahedron

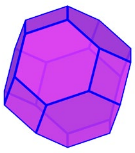

Truncated Octahedron

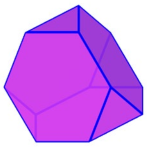

Truncated Tetrahedron

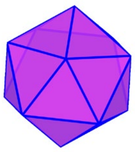

Gyroelongated Pentagonal Pyramid

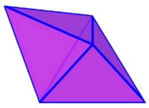

Triangular Bipyramid

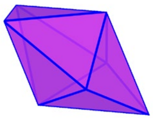

Pentagonal Bipyramid

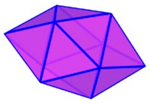

Gyroelongated Square Bipyramid

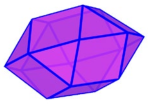

Square Gyrobicupola

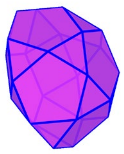

Pentagonal Orthocupolarotunda

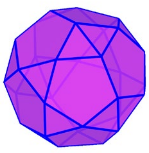

Pentagonal Orthobirotunda

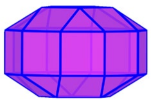

Elongated Pentagonal Gyrobicupola

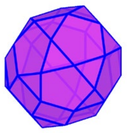

Elongated Pentagonal Gyrobirotunda

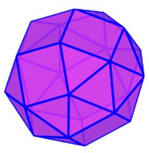

Gyroelongated Square Bicupola

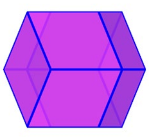

Rhombic Dodecahedron Dodecahedron

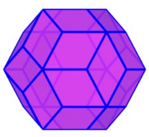

Rhombic Triacontahedron

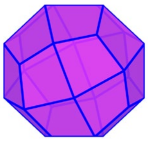

Deltoidal Icositetrahedron

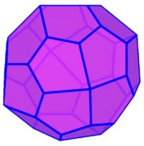

Pentagonal Icositetrahedron

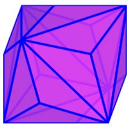

Triakis Octahedron

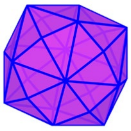

Disdyakis Dodecahedron

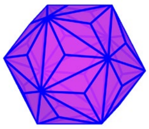

Triakis Icosahedron

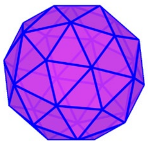

Pentakis Dodecahedron

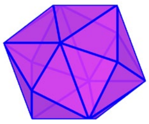

Tetrakis Hexahedron

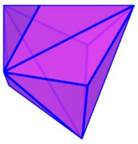

Triakis Tetrahedron

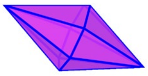

Heptagonal Bipyramid

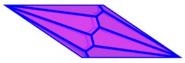

Enneagonal Trapezohedron

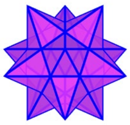

Small Stell Dodecahedron

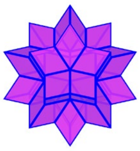

Rhombic Hexecontahedron

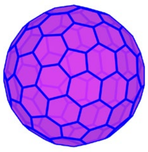

Goldberg Polyhedron dk5dgD

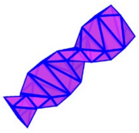

Double Helix

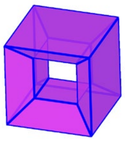

Nested Cube

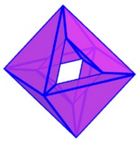

Nested Octahedron

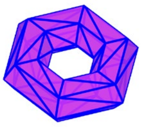

Torus

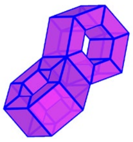

Double Torus

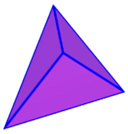

Asymmetric Tetrahedron

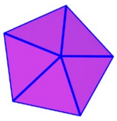

Pentagonal Pyramid

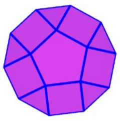

Pentagonal Cupola

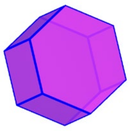

Hexagonal Prism

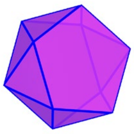

Pentagonal Antiprism

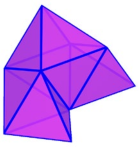

Chiral Object

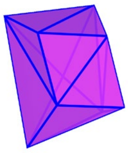

Twisted Triangular Prism

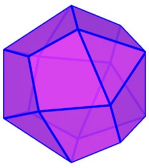

Biscribed Propello Tetrahedron

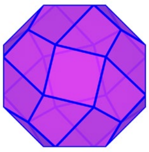

Biscribed Propello Cube

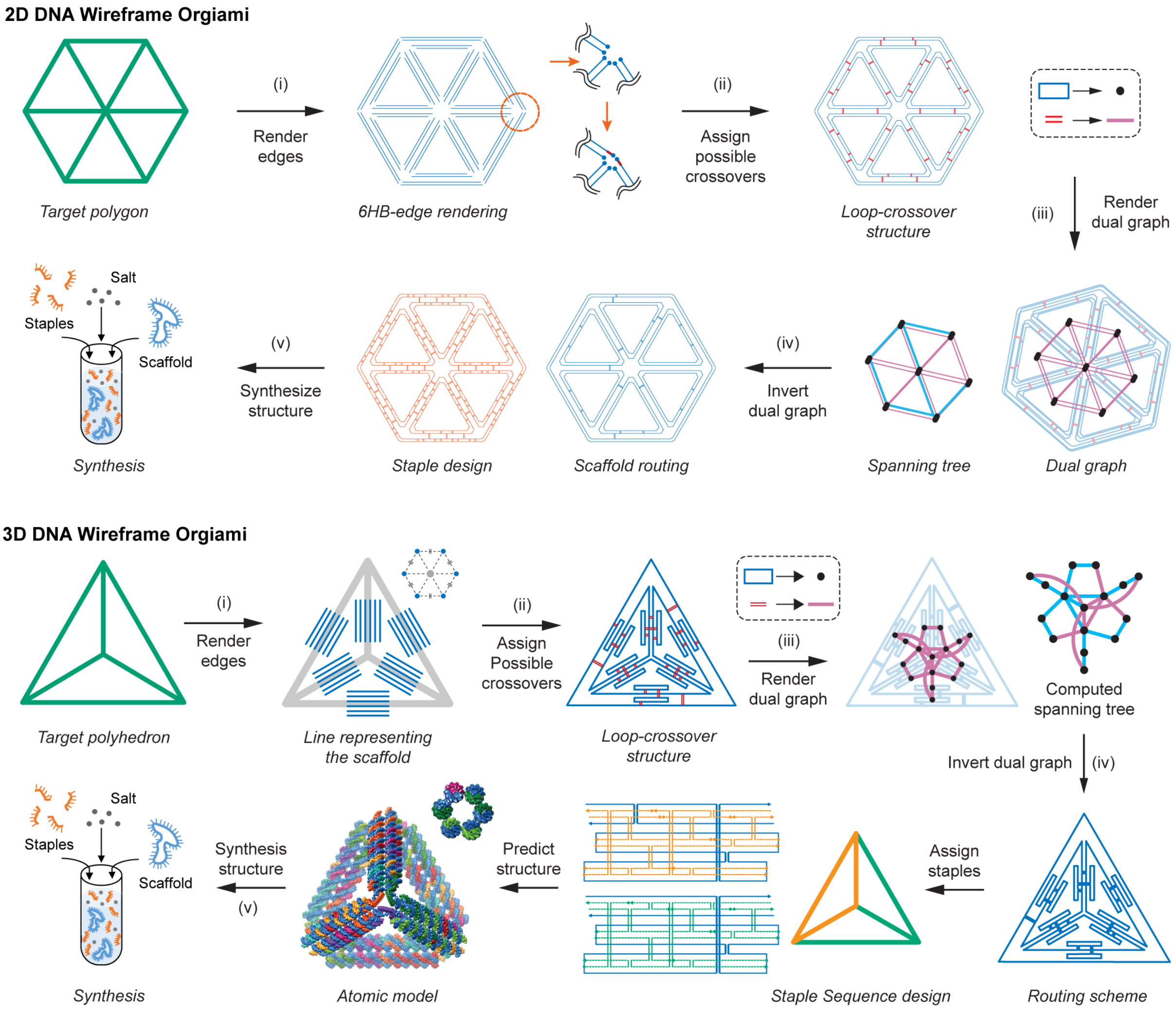

Figure S1. Design algorithm for 2D and 3D wireframe DNA origami. The arbitrary target 2D or 3D geometry is based on a polygonal mesh, with discretized line segments (step i) to represent two or six DNA duplexes per wireframe edge with the endpoints joined (step ii) to form closed loops with geometrically allowable scaffold double crossovers between them. The dual graph of the loop-crossover structure is obtained (step iii) by converting each closed scaffold loop to a node and each possible scaffold double crossover connecting them to an edge. The minimum spanning tree of the dual graph was then determined and inverted (step iv), defining the DNA scaffold routing. Staple sequences generated (step v) by the algorithm were used with the input scaffold to synthesize the 2D or 3D DNA origami in one-pot thermal annealing.

A

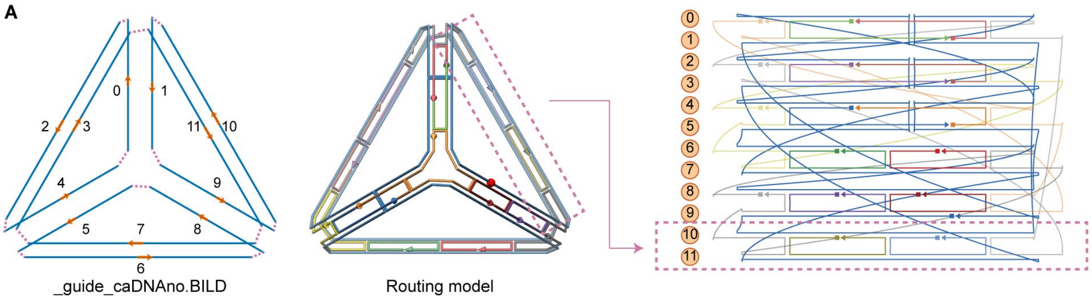

B

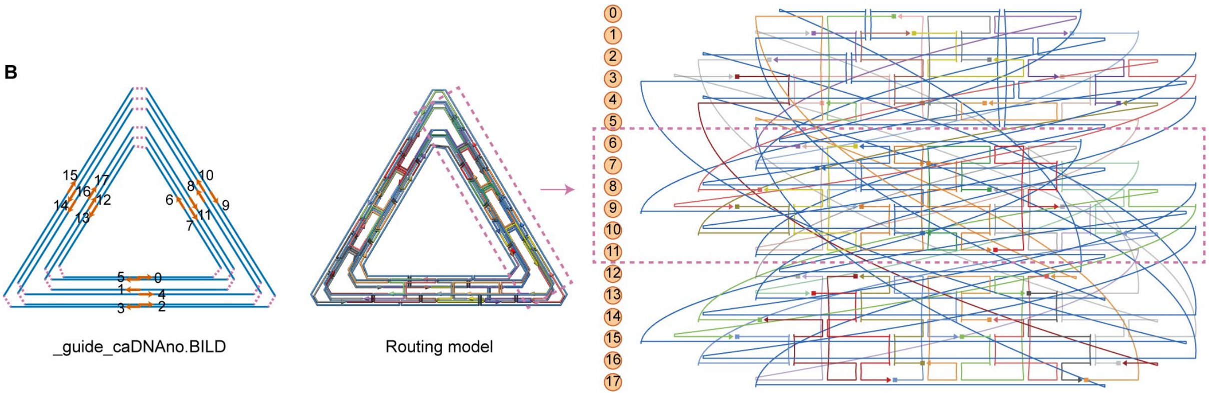

Figure S2. Exported SVG schematic of the DNA origami objects. (**A**) DX tetrahedron DNA origami with 63-bp edge-length (**B**) 6HB triangle DNA origami with 63-bp edge-length. Line numbers in the guide model (left) corresponds to row numbers in caDNAno and arrows (orange) indicate the direction of the scaffold. The red sphere in the routing model indicates the starting point of the single-stranded loop, whose position can be altered using caDNAno. caDNAno representation with scaffold (blue) and staple(multi-color) strands.

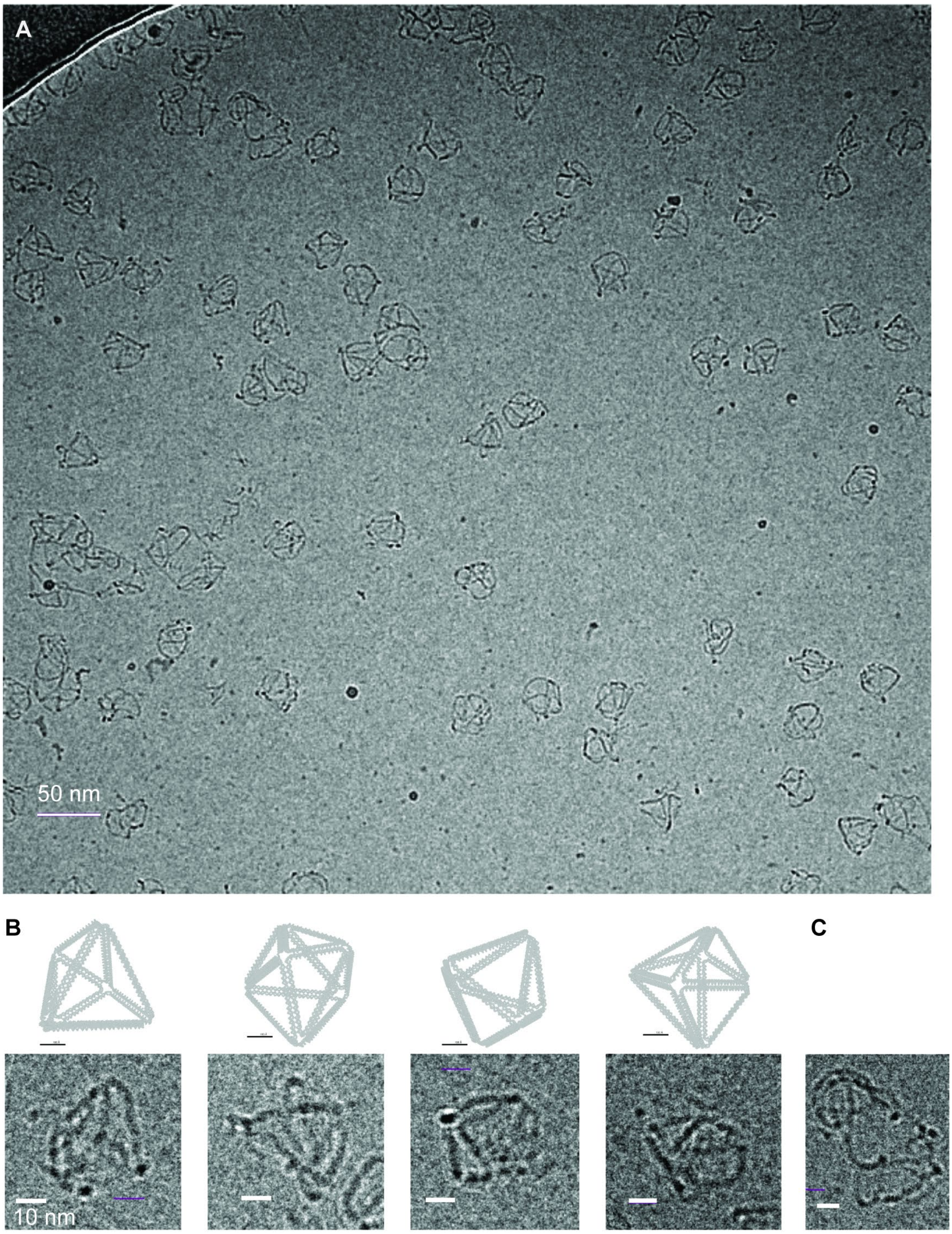

Figure S3. Cryo-EM imaging of DX-based asymmetric octahedral DNA origami of 63-bp edge-length, with continuous edge and arbitrary vertex angle design. (**A**) Representative micrograph at 73,000x magnification. (**B**) Atomic model of the origami viewed from various angles with matching particle images; all scale bars are 10 nm. (**C**) Example particle image of a dimerized DNA origami; scale bar is 10 nm.

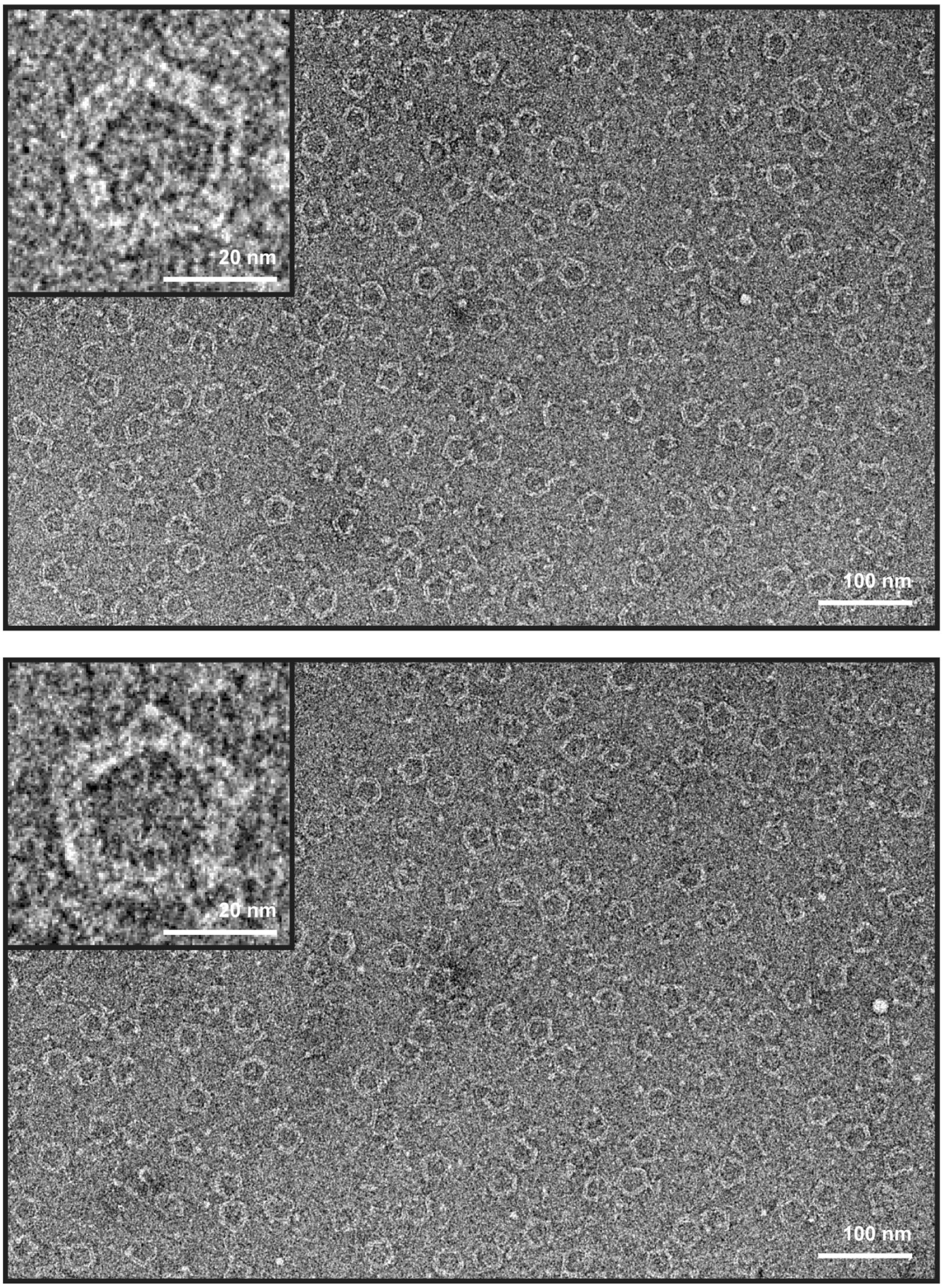

Figure S4. TEM imaging of 6HB-based pentagonal DNA origami of 42-bp edge-length without internal mesh.

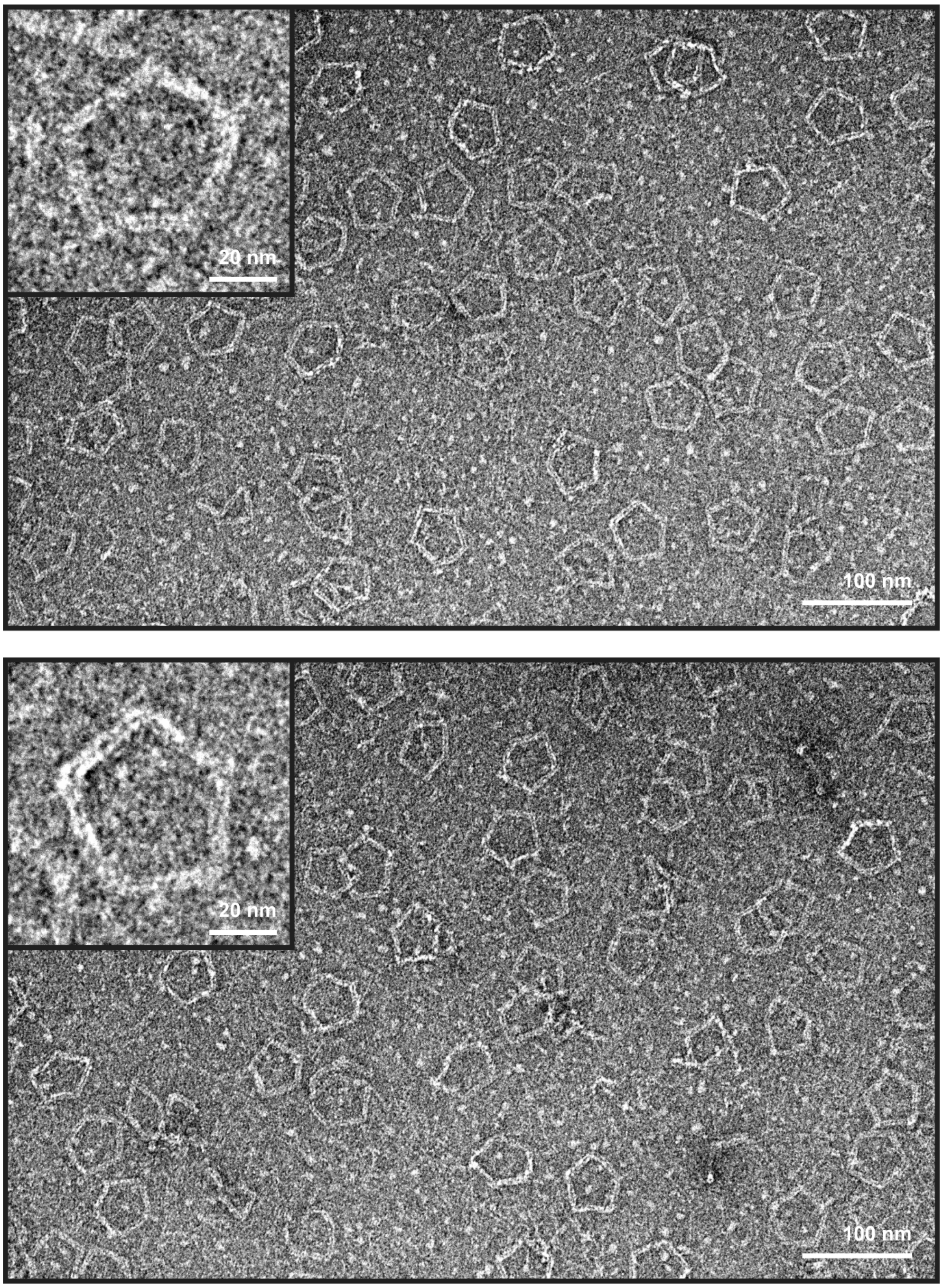

Figure S5. TEM imaging of 6HB-based pentagonal DNA origami of 84-bp edge-length without internal mesh.

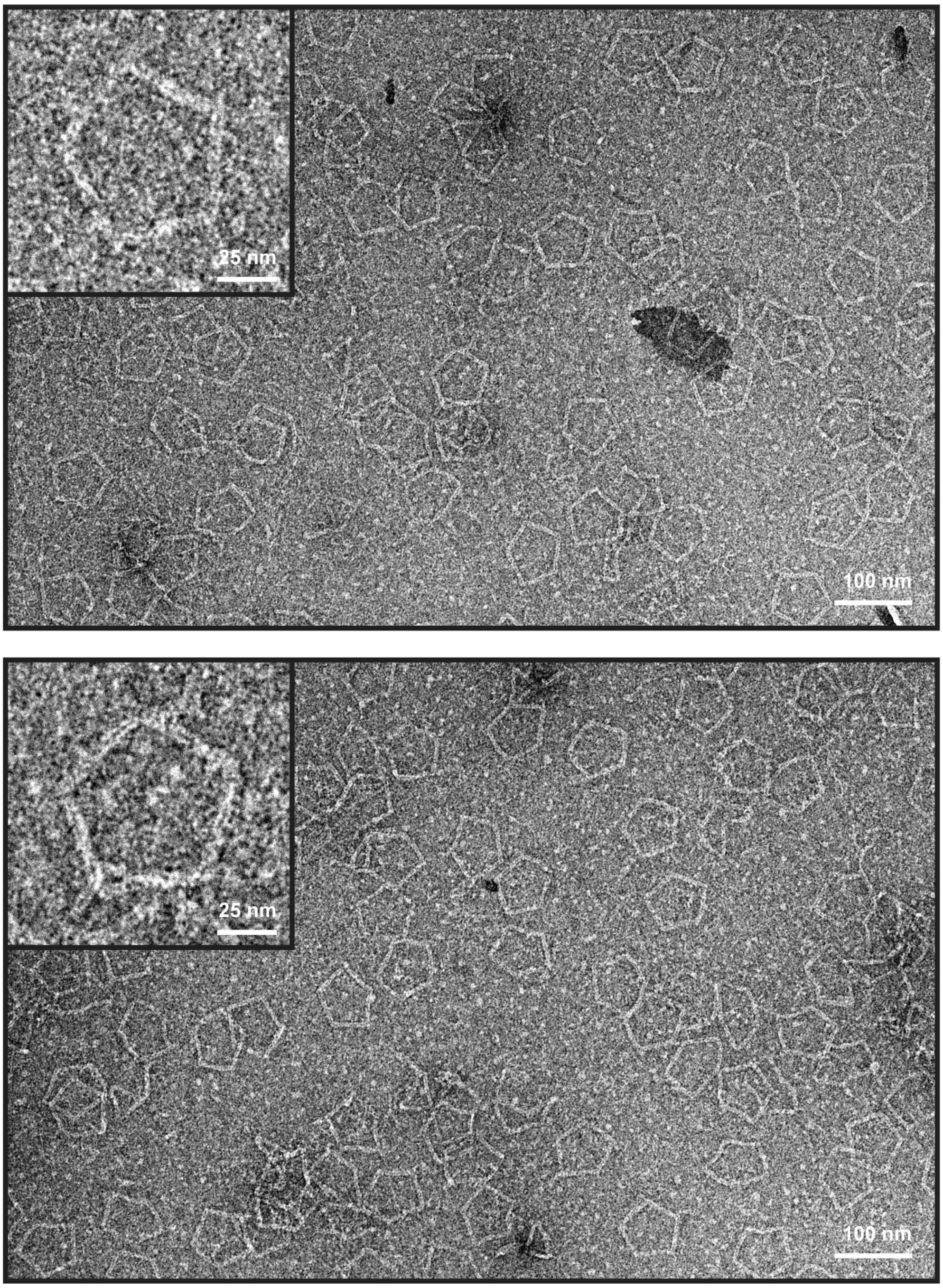

Figure S6. TEM imaging of 6HB-based pentagonal DNA origami of 126-bp edge-length without internal mesh.

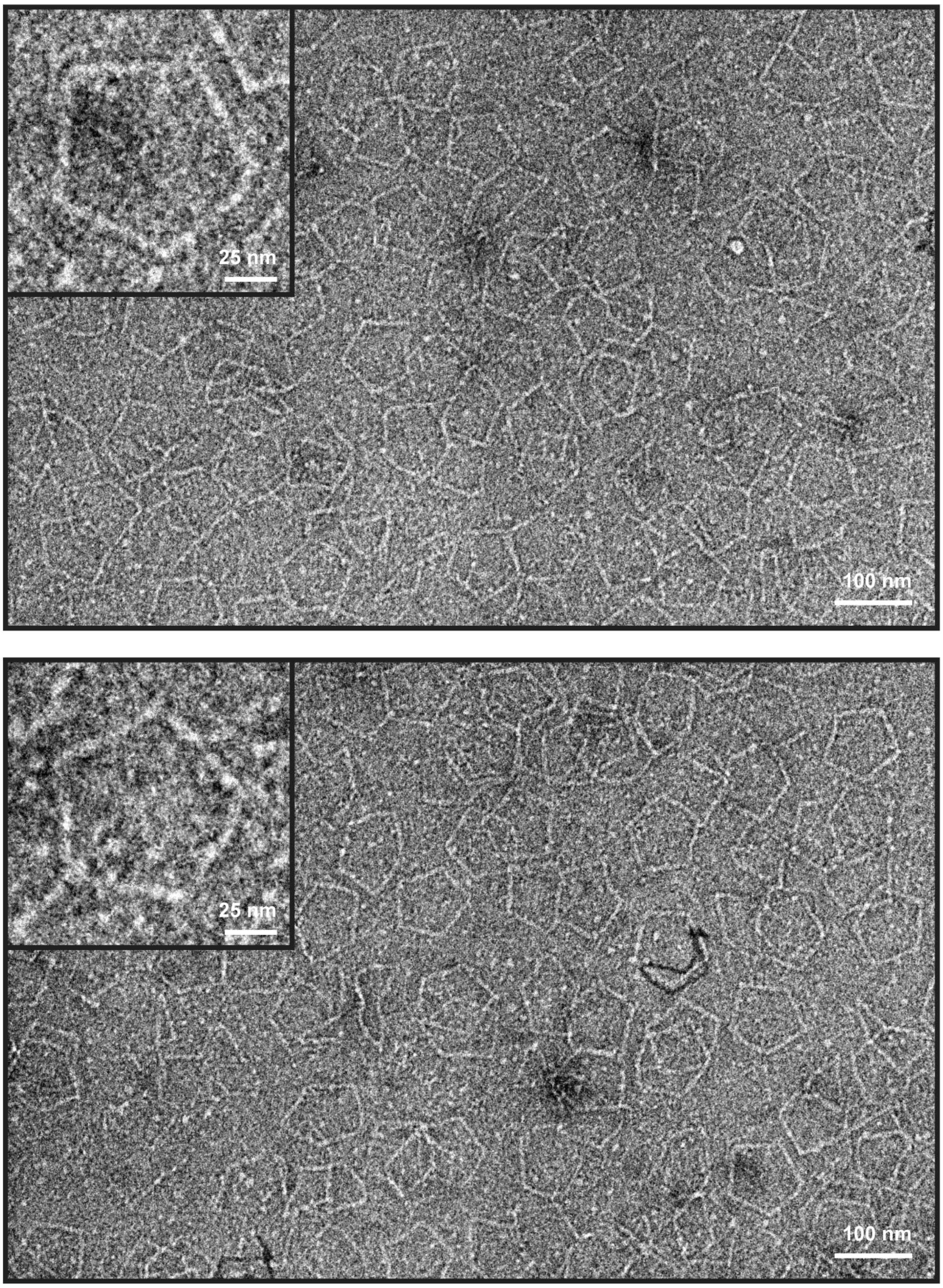

Figure S7. TEM imaging of 6HB-based pentagonal DNA origami of 168-bp edge-length without internal mesh.

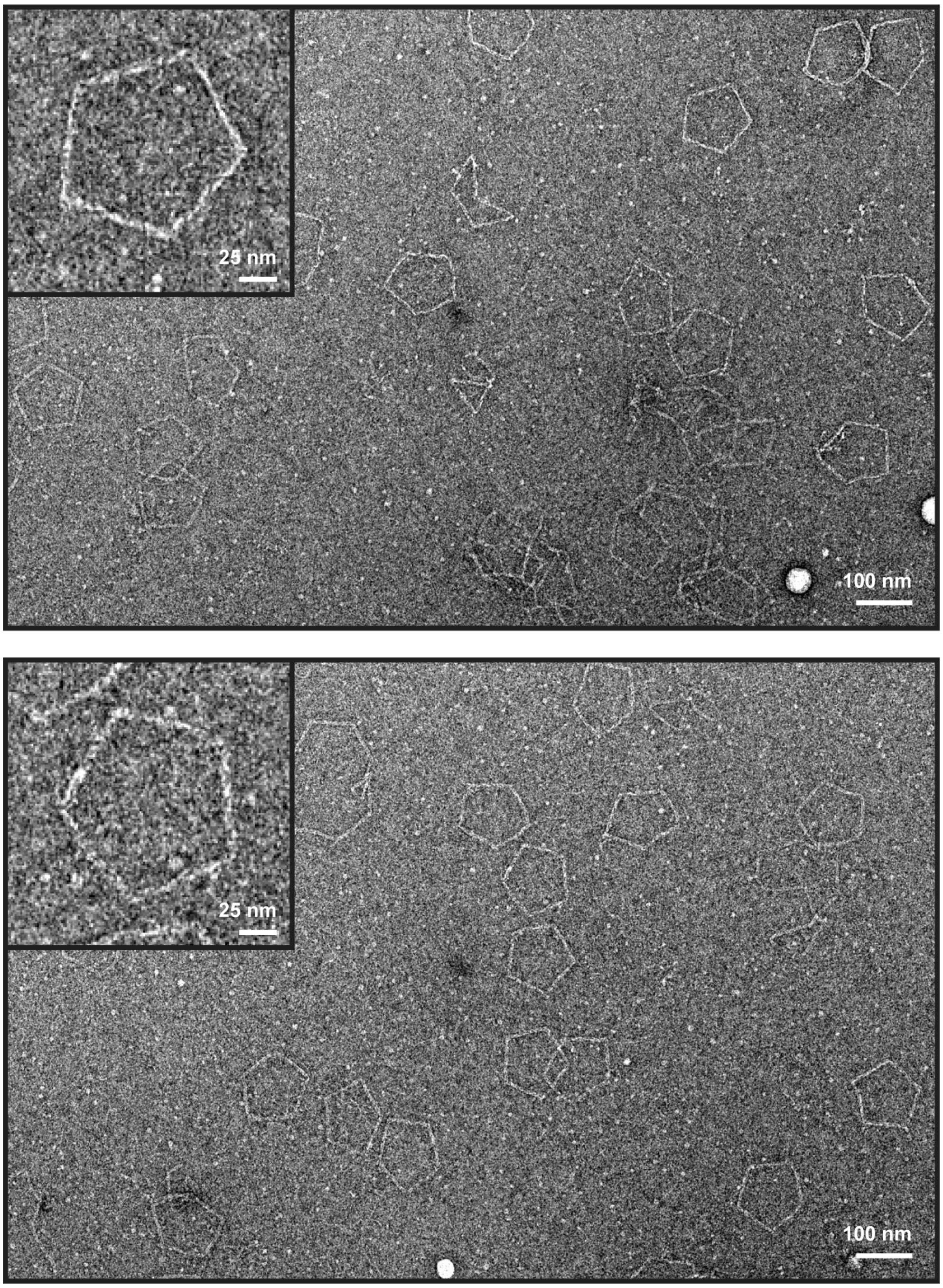

Figure S8. TEM imaging of 6HB-based pentagonal DNA origami of 210-bp edge-length without internal mesh.

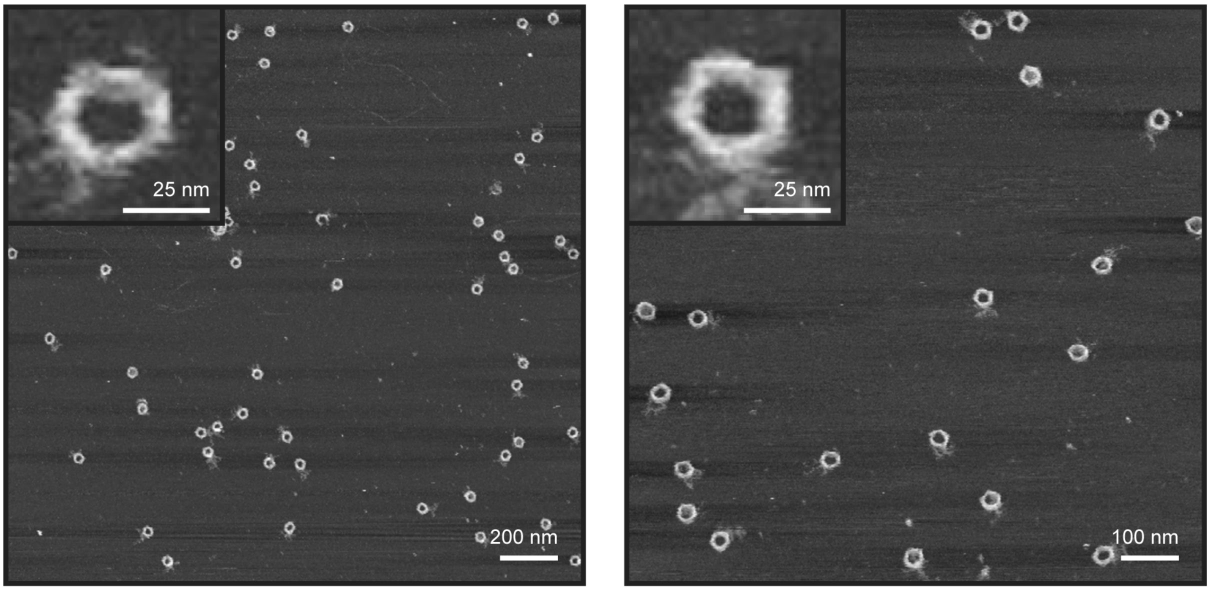

Figure S9. AFM imaging of 6HB-based pentagonal DNA origami of 42-bp edge-length without internal mesh.

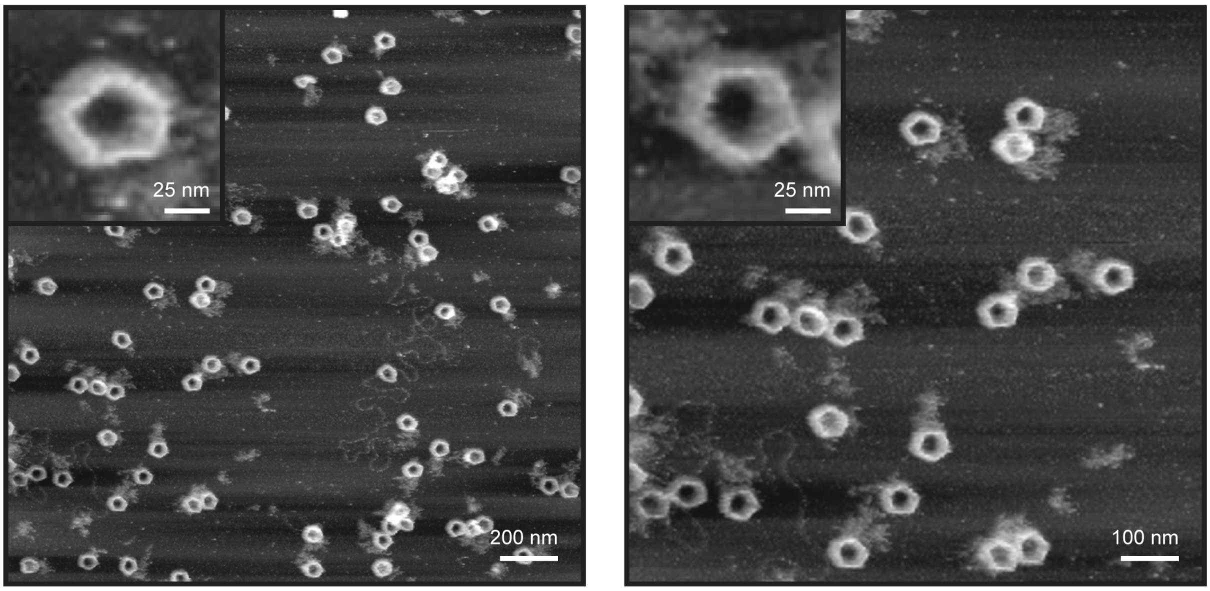

Figure S10. AFM imaging of 6HB-based pentagonal DNA origami of 84-bp edge-length without internal mesh.

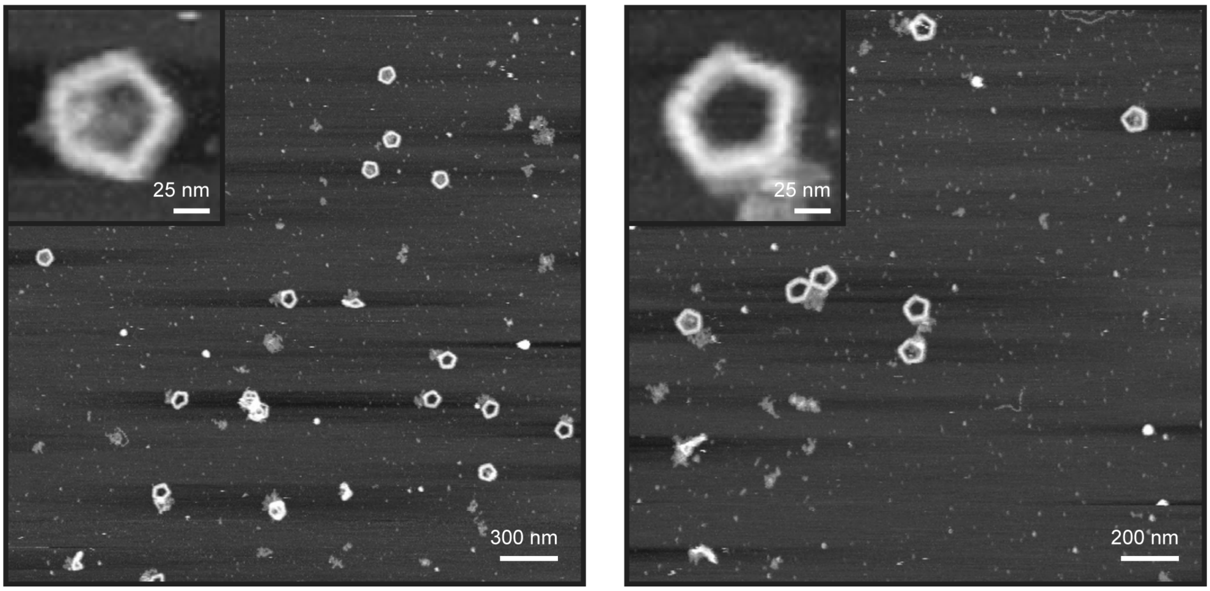

Figure S11. AFM imaging of 6HB-based pentagonal DNA origami of 126-bp edge-length without internal mesh.

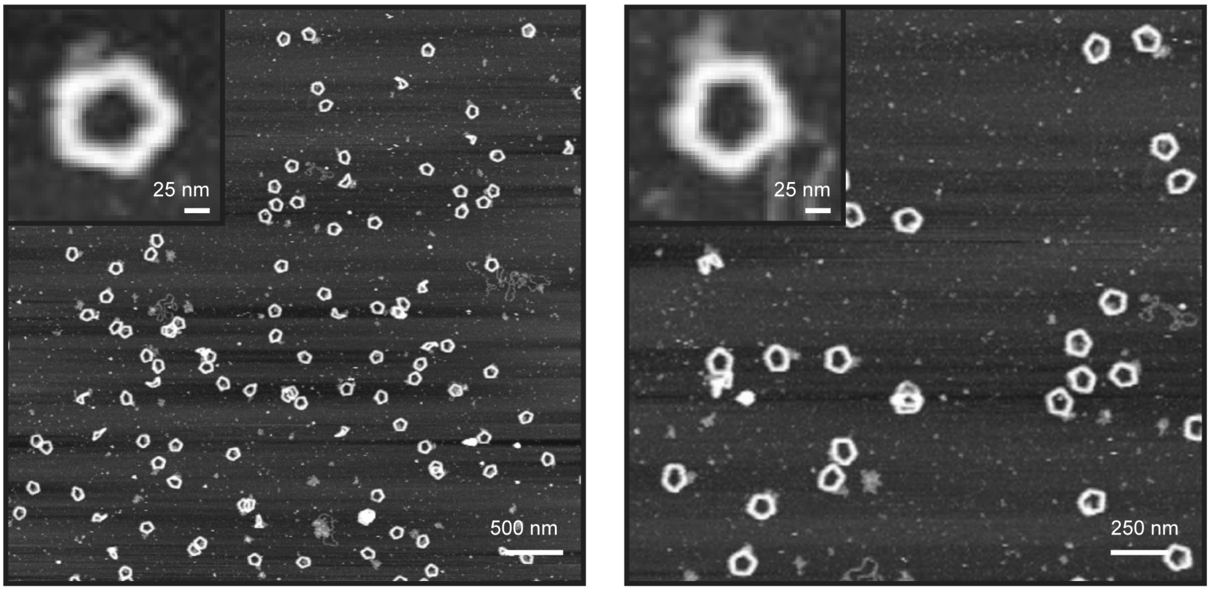

Figure S12. AFM imaging of 6HB-based pentagonal DNA origami of 168-bp edge-length without internal mesh.

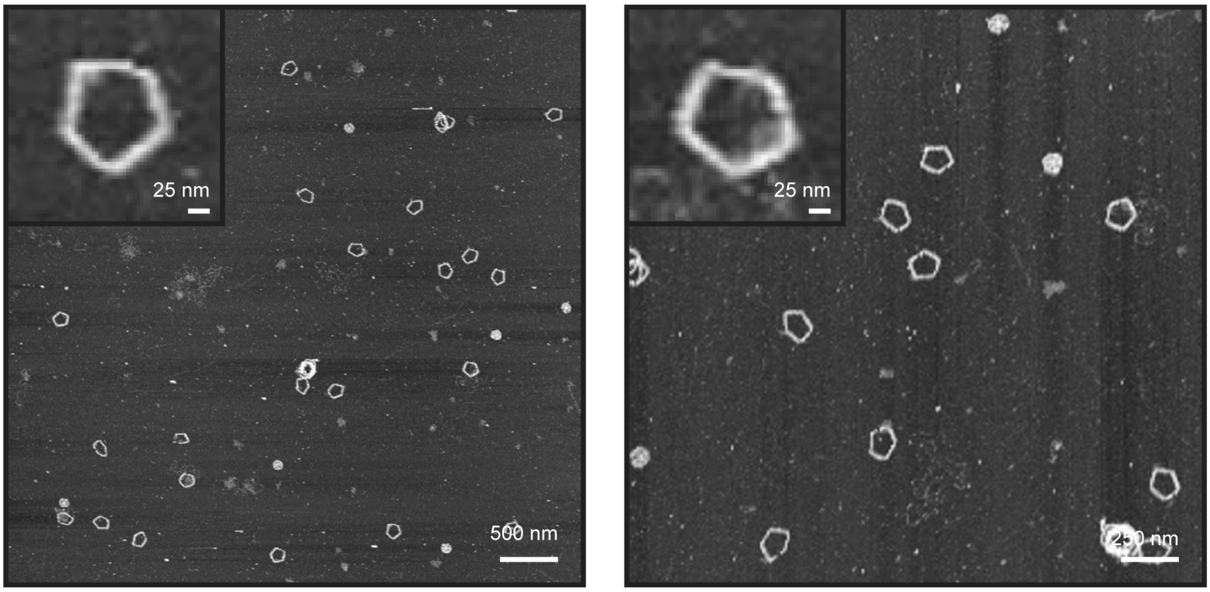

Figure S13. AFM imaging of 6HB-based pentagonal DNA origami of 210-bp edge-length without internal mesh.

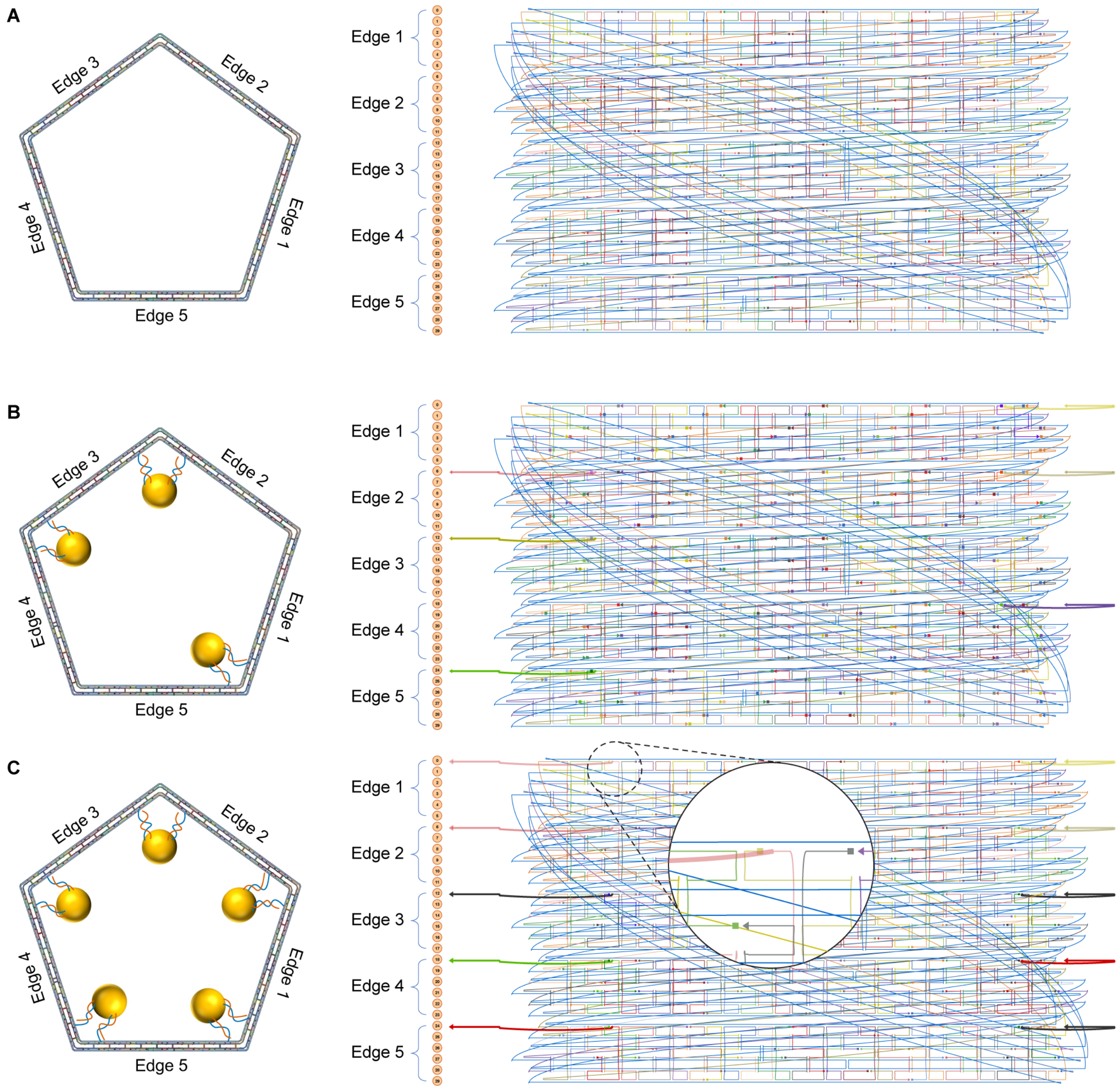

Figure S14. Editing origami objects for attaching nanogolds. (**A**) 6HB pentagonal DNA origami with 210-bp edge-length. 210-bp pentagonal DNA origami with extended staple strands for (**B**) three and (**C**) five nanogolds. caDNAno representation with scaffold (blue) and staple(multi-color) strands.

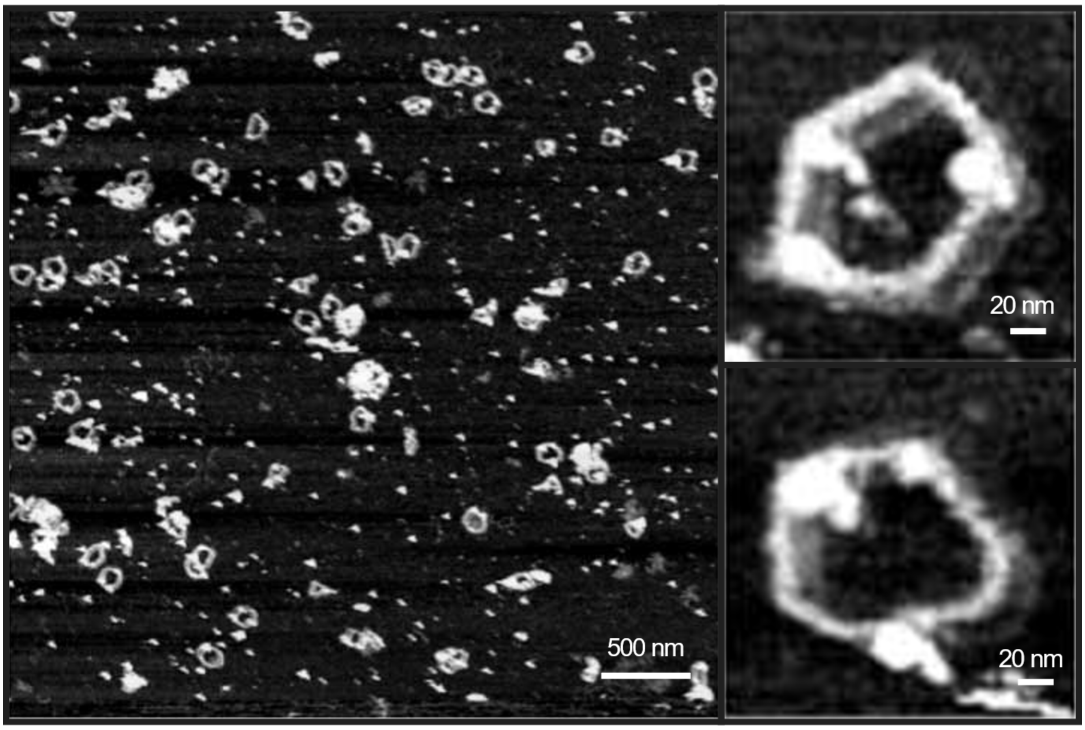

Figure S15. AFM imaging of 6HB-based pentagonal DNA origami of 210-bp edge-length three nanogolds.

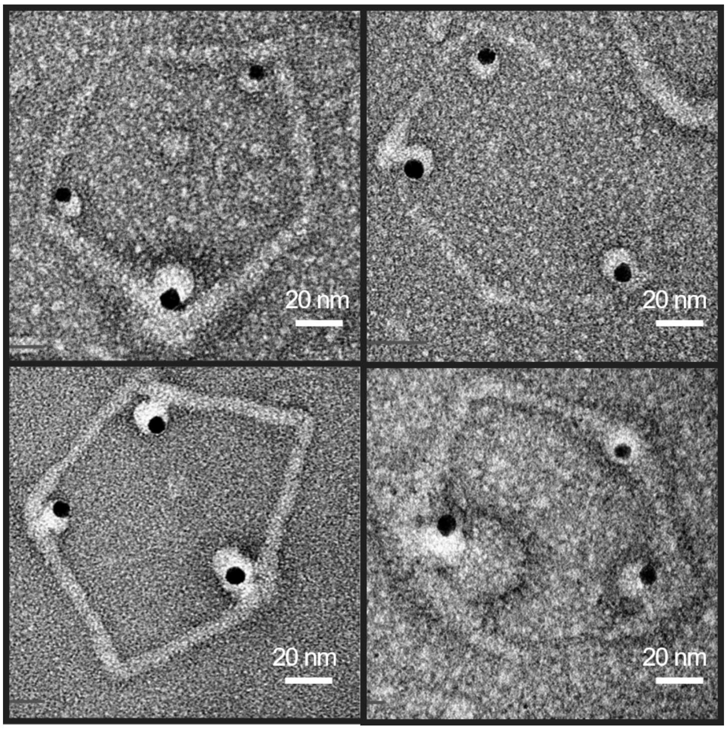

Figure S16. TEM imaging of 6HB-based pentagonal DNA origami of 210-bp edge-length with three nanogolds.

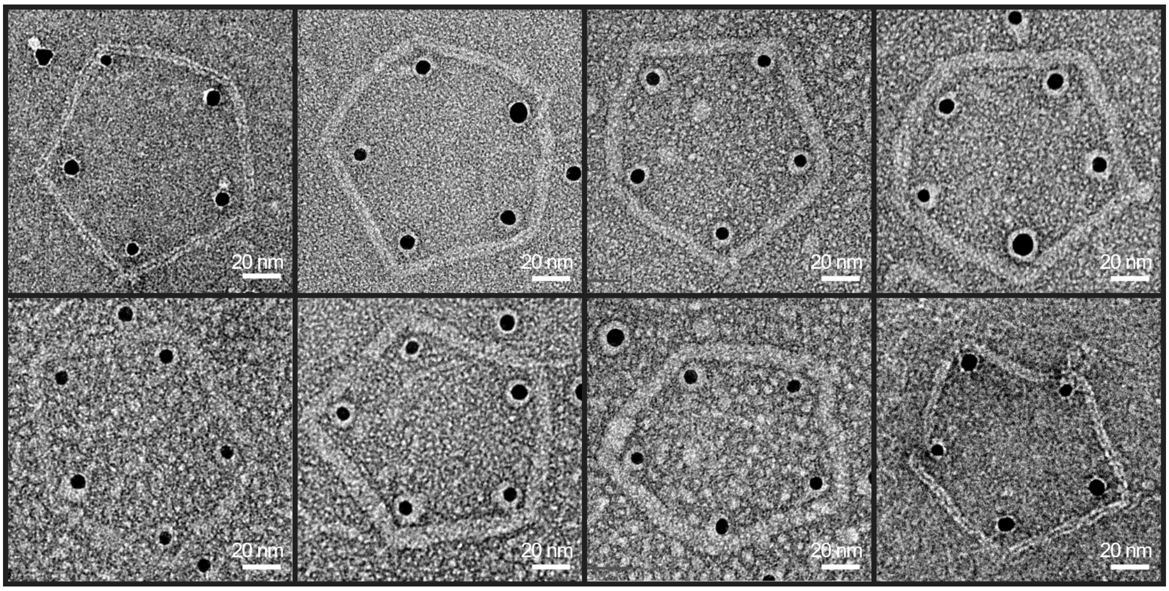

Figure S17. TEM imaging of 6HB-based pentagonal DNA origami of 210-bp edge-length with five nanogolds.

Table S1. Sequences for the 2,775-nt (#1), 7,249-nt (#2), and 2,520-nt (#3) length scaffolds used.

No	Scaffold sequence
#1	GAGCGCAACGCAATTAATGTGCGCCCTGTAGCGGCGCATTAAGCGCGGCGGGTGTGGTGGTTACGCGCAGCGTGACCGCTA<br>CACTTGCCAGCGCCCTAGCGCCCGCTCCTTTCGCTTTCTTCCCTTCCTTTCTCGCCACGTTCGCCGGCTTTCCCCGTCAAG<br>CTCTAAATCGGGGGCTCCCTTTAGGGTTCCGATTTAGTGCTTTACGGCACCTCGACCCCAAAAAACTTGATTAGGGTGATG<br>GTTCACGTAGTGGGCCATCGCCCTGATAGACGGTTTTTCGCCCTTTGACGTTGGAGTCCACGTTCTTTAATAGTGGACTCT<br>TGTTCCAAACTGGAACAACACTCAACCCTATCTCGGTCTATTCTTTTGATTTATAAGGGATTTTGCCGATTTCGGCCTATT<br>GGTTAAAAAATGAGCTGATTTAACAAAAATTTAACGCGAATTACAACCGGGGTACATATGATTGGGGTCTGACGCTCAGTG<br>GAACGAAAACTCACGTTAAGGGATTTTGGTCATGAGATTATCAAAAAGGATCTTCACCTAGATCCTTTTAAATTAAAAATG<br>AAGTTTTAAATCAATCTAAAGTATATATGAGTAAACTTGGTCTGACAGTTACCAATGCTTAATCAGTGAGGCACCTATCTC<br>AGCGATCTGTCTATTTCGTTCATCCATAGTTGCCTGACTCCCCGTCGTGTAGATAACTACGATACGGGAGGGCTTACCATC<br>TGGCCCCAGTGCTGCAATGATACCGCGAGACCCACGCTCACCGGCTCCAGATTTATCAGCAATAAACCAGCCAGCCGGAAG<br>GGCCGAGCGCAGAAGTGGTCCTGCAACTTTATCCGCCTCCATCCAGTCTATTAATTGTTGCCGGGAAGCTAGAGTAAGTAG<br>TTCGCCAGTTAATAGTTTGCGCAACGTTGTTGCCATTGCTACAGGCATCGTGGTGTCACGCTCGTCGTTTGGTATGGCTTC<br>ATTCAGCTCCGGTTCCCAACGATCAAGGCGAGTTACATGATCCCCCATGTTGTGCAAAAAAGCGGTTAGCTCCTTCGGTCC<br>TCCGATCGTTGTCAGAAGTAAGTTGGCCGCAGTGTTATCACTCATGGTTATGGCAGCACTGCATAATTCTCTTACTGTCAT<br>GCCATCCGTAAGATGCTTTTCTGTGACTGGTGAGTACTCAACCAAGTCATTCTGAGAATAGTGTATGCGGCGACCGAGTTG<br>CTCTTGCCCGGCGTCAATACGGGATAATACCGCGCCACATAGCAGAACTTTAAAAGTGCTCATCATTGGAAAACGTTCTTC<br>GGGGCGAAAACTCTCAAGGATCTTACCGCTGTTGAGATCCAGTTCGATGTAACCCACTCGTGCACCCAACTGATCTTCAGC<br>ATCTTTTACTTTCACCAGCGTTTCTGGGTGAGCAAAAACAGGAAGGCAAAATGCCGCAAAAAAGGGAATAAGGGCGACACG<br>GAAATGTTGAATACTCATACTCTTCCTTTTTCAATATTATTGAAGCATTTATCAGGGTTATTGTCTCATGAGCGGATACAT<br>ATTTGAATGTATTTAGAAAAATAAACAAATAGGGGTTCCGCGCACATTTCCCCGAAAAGTGCCACCTGACGTCTAAGAAAC<br>CATTATTATCATGACATTAACCTATAAAAATAGGCGTATCACGAGGCCCTTTCGTCGAATTCGTCGTCGTCCCCTCAAACT<br>CTTGGGTGGAGAGGCTATTCGTTTAAGGTCACATCGCATGTAATTTACTTATTCTCTGTTGTTGAGCCACCCGGGCGCCAG<br>ATTTTGTTTAAAGCTTTGTCTCTTAGTTTGTATAGACAGATTCAGAGTGCAAGGTTTCGTTCGCTCGTACCTGGTTTTCCC<br>TGGTTCTTCACAGATAGGATTTGACTTTCTACAACACTTATGCGGCTTCCTACCCGTTTGAAGGCCGATACAGGTGCTGCG

	CAAAATGCGGGCGAACATAGAGTATCAAAACAACGCCTTCTAATCTAGGAATATAGGGAAGATACGTATTTGCTACCATGC<br>TTTCTGGGGTCATTAACGACCAACCTCTTTTCTTTTAAAGTAGGATTGCACAATGAATGAATACACGTGGTCCGATAACTG<br>ACCAAGTAACATGGTTATCACTCGATGTCCGCCAGACGTGTGCAAACCAACCCGGGAGTTACGTCACTAATCCTTCGCTAC<br>GTCGTGAAGATATTTACTTGTGAATATCGAGGGTAATAAGATAATAGACTGTGACTAGTATTGCCAGACTGTCGCTACCTG<br>CAACACATAACTATCCTGAGGTTACTGCATAGTACTGATTACACCCGAGTCAAAATTTCTAACTTCTAACATGTACCTAGT<br>AACCAGCTCAATAATTATGTCAGAATATAGCTCTGGGAACCCTCGGACAATTATGATACACGGTATTAATATCTTGCTTGC<br>GTTAGCCACTTCTCATCTTTGGATACCGATTCTATTTTGCATAGCAGTTCCTTTTACACATATAAGAATTTCGCCATAGGT<br>ATGACCTACCCCAGATCGTCGATTATCTGCTGGAAAATTTATTTAACACTATGTTTCTCTCCAGATGTGAGTATACACGAT<br>AAATAATACCTGGGTACCGGTTGGTGTTATTACCTTGTTTCTAAGTGCTTAATCGGCGCTTAGTGATAAGGTTGTACTAGT<br>CGACGCGTGGCCGCCAATTATTCTTGTCATAATTTGACTTTGTTCTATATGACTATGATCTCCTGTCATCTCACCTATTGA<br>TGCCACCTTTTCAGCCTGCAG
**#2**	AATGCTACTACTATTAGTAGAATTGATGCCACCTTTTCAGCTCGCGCCCCAAATGAAAATATAGCTAAACAGGTTATTGAC<br>CATTTGCGAAATGTATCTAATGGTCAAACTAAATCTACTCGTTCGCAGAATTGGGAATCAACTGTTATATGGAATGAAACT<br>TCCAGACACCGTACTTTAGTTGCATATTTAAAACATGTTGAGCTACAGCATTATATTCAGCAATTAAGCTCTAAGCCATCC<br>GCAAAAATGACCTCTTATCAAAAGGAGCAATTAAAGGTACTCTCTAATCCTGACCTGTTGGAGTTTGCTTCCGGTCTGGTT<br>CGCTTTGAAGCTCGAATTAAAACGCGATATTTGAAGTCTTTCGGGCTTCCTCTTAATCTTTTTGATGCAATCCGCTTTGCT<br>TCTGACTATAATAGTCAGGGTAAAGACCTGATTTTTGATTTATGGTCATTCTCGTTTTCTGAACTGTTTAAAGCATTTGAG<br>GGGGATTCAATGAATATTTATGACGATTCCGCAGTATTGGACGCTATCCAGTCTAAACATTTTACTATTACCCCCTCTGGC<br>AAAACTTCTTTTGCAAAAGCCTCTCGCTATTTTGGTTTTTATCGTCGTCTGGTAAACGAGGGTTATGATAGTGTTGCTCTT<br>ACTATGCCTCGTAATTCCTTTTGGCGTTATGTATCTGCATTAGTTGAATGTGGTATTCCTAAATCTCAACTGATGAATCTT<br>TCTACCTGTAATAATGTTGTTCCGTTAGTTCGTTTTATTAACGTAGATTTTTCTTCCCAACGTCCTGACTGGTATAATGAG<br>CCAGTTCTTAAAATCGCATAAGGTAATTCACAATGATTAAAGTTGAAATTAAACCATCTCAAGCCCAATTTACTACTCGTT<br>CTGGTGTTTCTCGTCAGGGCAAGCCTTATTCACTGAATGAGCAGCTTTGTTACGTTGATTTGGGTAATGAATATCCGGTTC<br>TTGTCAAGATTACTCTTGATGAAGGTCAGCCAGCCTATGCGCCTGGTCTGTACACCGTTCATCTGTCCTCTTTCAAAGTTG<br>GTCAGTTCGGTTCCCTTATGATTGACCGTCTGCGCCTCGTTCCGGCTAAGTAACATGGAGCAGGTCGCGGATTTCGACACA<br>ATTTATCAGGCGATGATACAAATCTCCGTTGTACTTTGTTTCGCGCTTGGTATAATCGCTGGGGGTCAAAGATGAGTGTTT<br>TAGTGTATTCTTTTGCCTCTTTCGTTTTAGGTTGGTGCCTTCGTAGTGGCATTACGTATTTTACCCGTTTAATGGAAACTT<br>CCTCATGAAAAAGTCTTTAGTCCTCAAAGCCTCTGTAGCCGTTGCTACCCTCGTTCCGATGCTGTCTTTCGCTGCTGAGGG<br>TGACGATCCCGCAAAAGCGGCCTTTAACTCCCTGCAAGCCTCAGCGACCGAATATATCGGTTATGCGTGGGCGATGGTTGT<br>TGTCATTGTCGGCGCAACTATCGGTATCAAGCTGTTTAAGAAATTCACCTCGAAAGCAAGCTGATAAACCGATACAATTAA<br>AGGCTCCTTTTGGAGCCTTTTTTTTGGAGATTTTCAACGTGAAAAAATTATTATTCGCAATTCCTTTAGTTGTTCCTTTCT<br>ATTCTCACTCCGCTGAAACTGTTGAAAGTTGTTTAGCAAAATCCCATACAGAAAATTCATTTACTAACGTCTGGAAAGACG<br>ACAAAACTTTAGATCGTTACGCTAACTATGAGGGCTGTCTGTGGAATGCTACAGGCGTTGTAGTTTGTACTGGTGACGAAA<br>CTCAGTGTTACGGTACATGGGTTCCTATTGGGCTTGCTATCCCTGAAAATGAGGGTGGTGGCTCTGAGGGTGGCGGTTCTG<br>AGGGTGGCGGTTCTGAGGGTGGCGGTACTAAACCTCCTGAGTACGGTGATACACCTATTCCGGGCTATACTTATATCAACC<br>CTCTCGACGGCACTTATCCGCCTGGTACTGAGCAAAACCCCGCTAATCCTAATCCTTCTCTTGAGGAGTCTCAGCCTCTTA<br>ATACTTTCATGTTTCAGAATAATAGGTTCCGAAATAGGCAGGGGGCATTAACTGTTTATACGGGCACTGTTACTCAAGGCA<br>CTGACCCCGTTAAAACTTATTACCAGTACACTCCTGTATCATCAAAAGCCATGTATGACGCTTACTGGAACGGTAAATTCA<br>GAGACTGCGCTTTCCATTCTGGCTTTAATGAGGATTTATTTGTTTGTGAATATCAAGGCCAATCGTCTGACCTGCCTCAAC<br>CTCCTGTCAATGCTGGCGGCGGCTCTGGTGGTGGTTCTGGTGGCGGCTCTGAGGGTGGTGGCTCTGAGGGTGGCGGTTCTG<br>AGGGTGGCGGCTCTGAGGGAGGCGGTTCCGGTGGTGGCTCTGGTTCCGGTGATTTTGATTATGAAAAGATGGCAAACGCTA<br>ATAAGGGGGCTATGACCGAAAATGCCGATGAAAACGCGCTACAGTCTGACGCTAAAGGCAAACTTGATTCTGTCGCTACTG<br>ATTACGGTGCTGCTATCGATGGTTTCATTGGTGACGTTTCCGGCCTTGCTAATGGTAATGGTGCTACTGGTGATTTTGCTG<br>GCTCTAATTCCCAAATGGCTCAAGTCGGTGACGGTGATAATTCACCTTTAATGAATAATTTCCGTCAATATTTACCTTCCC<br>TCCCTCAATCGGTTGAATGTCGCCCTTTTGTCTTTGGCGCTGGTAAACCATATGAATTTTCTATTGATTGTGACAAAATAA<br>ACTTATTCCGTGGTGTCTTTGCGTTTCTTTTATATGTTGCCACCTTTATGTATGTATTTTCTACGTTTGCTAACATACTGC<br>GTAATAAGGAGTCTTAATCATGCCAGTTCTTTTGGGTATTCCGTTATTATTGCGTTTCCTCGGTTTCCTTCTGGTAACTTT<br>GTTCGGCTATCTGCTTACTTTTCTTAAAAAGGGCTTCGGTAAGATAGCTATTGCTATTTCATTGTTTCTTGCTCTTATTAT<br>TGGGCTTAACTCAATTCTTGTGGGTTATCTCTCTGATATTAGCGCTCAATTACCCTCTGACTTTGTTCAGGGTGTTCAGTT<br>AATTCTCCCGTCTAATGCGCTTCCCTGTTTTTATGTTATTCTCTCTGTAAAGGCTGCTATTTTCATTTTTGACGTTAAACA<br>AAAAATCGTTTCTTATTTGGATTGGGATAAATAATATGGCTGTTTATTTTGTAACTGGCAAATTAGGCTCTGGAAAGACGC<br>TCGTTAGCGTTGGTAAGATTCAGGATAAAATTGTAGCTGGGTGCAAAATAGCAACTAATCTTGATTTAAGGCTTCAAAACC<br>TCCCGCAAGTCGGGAGGTTCGCTAAAACGCCTCGCGTTCTTAGAATACCGGATAAGCCTTCTATATCTGATTTGCTTGCTA<br>TTGGGCGCGGTAATGATTCCTACGATGAAAATAAAAACGGCTTGCTTGTTCTCGATGAGTGCGGTACTTGGTTTAATACCC<br>GTTCTTGGAATGATAAGGAAAGACAGCCGATTATTGATTGGTTTCTACATGCTCGTAAATTAGGATGGGATATTATTTTTC<br>TTGTTCAGGACTTATCTATTGTTGATAAACAGGCGCGTTCTGCATTAGCTGAACATGTTGTTTATTGTCGTCGTCTGGACA

	GAATTACTTTACCTTTTGTCGGTACTTTATATTCTCTTATTACTGGCTCGAAAATGCCTCTGCCTAAATTACATGTTGGCG<br>TTGTTAAATATGGCGATTCTCAATTAAGCCCTACTGTTGAGCGTTGGCTTTATACTGGTAAGAATTTGTATAACGCATATG<br>ATACTAAACAGGCTTTTTCTAGTAATTATGATTCCGGTGTTTATTCTTATTTAACGCCTTATTTATCACACGGTCGGTATT<br>TCAAACCATTAAATTTAGGTCAGAAGATGAAATTAACTAAAATATATTTGAAAAAGTTTTCTCGCGTTCTTTGTCTTGCGA<br>TTGGATTTGCATCAGCATTTACATATAGTTATATAACCCAACCTAAGCCGGAGGTTAAAAAGGTAGTCTCTCAGACCTATG<br>ATTTTGATAAATTCACTATTGACTCTTCTCAGCGTCTTAATCTAAGCTATCGCTATGTTTTCAAGGATTCTAAGGGAAAAT<br>TAATTAATAGCGACGATTTACAGAAGCAAGGTTATTCACTCACATATATTGATTTATGTACTGTTTCCATTAAAAAAGGTA<br>ATTCAAATGAAATTGTTAAATGTAATTAATTTTGTTTTCTTGATGTTTGTTTCATCATCTTCTTTTGCTCAGGTAATTGAA<br>ATGAATAATTCGCCTCTGCGCGATTTTGTAACTTGGTATTCAAAGCAATCAGGCGAATCCGTTATTGTTTCTCCCGATGTA<br>AAAGGTACTGTTACTGTATATTCATCTGACGTTAAACCTGAAAATCTACGCAATTTCTTTATTTCTGTTTTACGTGCAAAT<br>AATTTTGATATGGTAGGTTCTAACCCTTCCATTATTCAGAAGTATAATCCAAACAATCAGGATTATATTGATGAATTGCCA<br>TCATCTGATAATCAGGAATATGATGATAATTCCGCTCCTTCTGGTGGTTTCTTTGTTCCGCAAAATGATAATGTTACTCAA<br>ACTTTTAAAATTAATAACGTTCGGGCAAAGGATTTAATACGAGTTGTCGAATTGTTTGTAAAGTCTAATACTTCTAAATCC<br>TCAAATGTATTATCTATTGACGGCTCTAATCTATTAGTTGTTAGTGCTCCTAAAGATATTTTAGATAACCTTCCTCAATTC<br>CTTTCAACTGTTGATTTGCCAACTGACCAGATATTGATTGAGGGTTTGATATTTGAGGTTCAGCAAGGTGATGCTTTAGAT<br>TTTTCATTTGCTGCTGGCTCTCAGCGTGGCACTGTTGCAGGCGGTGTTAATACTGACCGCCTCACCTCTGTTTTATCTTCT<br>GCTGGTGGTTCGTTCGGTATTTTTAATGGCGATGTTTTAGGGCTATCAGTTCGCGCATTAAAGACTAATAGCCATTCAAAA<br>ATATTGTCTGTGCCACGTATTCTTACGCTTTCAGGTCAGAAGGGTTCTATCTCTGTTGGCCAGAATGTCCCTTTTATTACT<br>GGTCGTGTGACTGGTGAATCTGCCAATGTAAATAATCCATTTCAGACGATTGAGCGTCAAAATGTAGGTATTTCCATGAGC<br>GTTTTTCCTGTTGCAATGGCTGGCGGTAATATTGTTCTGGATATTACCAGCAAGGCCGATAGTTTGAGTTCTTCTACTCAG<br>GCAAGTGATGTTATTACTAATCAAAGAAGTATTGCTACAACGGTTAATTTGCGTGATGGACAGACTCTTTTACTCGGTGGC<br>CTCACTGATTATAAAAACACTTCTCAGGATTCTGGCGTACCGTTCCTGTCTAAAATCCCTTTAATCGGCCTCCTGTTTAGC<br>TCCCGCTCTGATTCTAACGAGGAAAGCACGTTATACGTGCTCGTCAAAGCAACCATAGTACGCGCCCTGTAGCGGCGCATT<br>AAGCGCGGCGGGTGTGGTGGTTACGCGCAGCGTGACCGCTACACTTGCCAGCGCCCTAGCGCCCGCTCCTTTCGCTTTCTT<br>CCCTTCCTTTCTCGCCACGTTCGCCGGCTTTCCCCGTCAAGCTCTAAATCGGGGGCTCCCTTTAGGGTTCCGATTTAGTGC<br>TTTACGGCACCTCGACCCCAAAAAACTTGATTTGGGTGATGGTTCACGTAGTGGGCCATCGCCCTGATAGACGGTTTTTCG<br>CCCTTTGACGTTGGAGTCCACGTTCTTTAATAGTGGACTCTTGTTCCAAACTGGAACAACACTCAACCCTATCTCGGGCTA<br>TTCTTTTGATTTATAAGGGATTTTGCCGATTTCGGAACCACCATCAAACAGGATTTTCGCCTGCTGGGGCAAACCAGCGTG<br>GACCGCTTGCTGCAACTCTCTCAGGGCCAGGCGGTGAAGGGCAATCAGCTGTTGCCCGTCTCACTGGTGAAAAGAAAAACC<br>ACCCTGGCGCCCAATACGCAAACCGCCTCTCCCCGCGCGTTGGCCGATTCATTAATGCAGCTGGCACGACAGGTTTCCCGA<br>CTGGAAAGCGGGCAGTGAGCGCAACGCAATTAATGTGAGTTAGCTCACTCATTAGGCACCCCAGGCTTTACACTTTATGCT<br>TCCGGCTCGTATGTTGTGTGGAATTGTGAGCGGATAACAATTTCACACAGGAAACAGCTATGACCATGATTACGAATTCGA<br>GCTCGGTACCCGGGGATCCTCTAGAGTCGACCTGCAGGCATGCAAGCTTGGCACTGGCCGTCGTTTTACAACGTCGTGACT<br>GGGAAAACCCTGGCGTTACCCAACTTAATCGCCTTGCAGCACATCCCCCTTTCGCCAGCTGGCGTAATAGCGAAGAGGCCC<br>GCACCGATCGCCCTTCCCAACAGTTGCGCAGCCTGAATGGCGAATGGCGCTTTGCCTGGTTTCCGGCACCAGAAGCGGTGC<br>CGGAAAGCTGGCTGGAGTGCGATCTTCCTGAGGCCGATACTGTCGTCGTCCCCTCAAACTGGCAGATGCACGGTTACGATG<br>CGCCCATCTACACCAACGTGACCTATCCCATTACGGTCAATCCGCCGTTTGTTCCCACGGAGAATCCGACGGGTTGTTACT<br>CGCTCACATTTAATGTTGATGAAAGCTGGCTACAGGAAGGCCAGACGCGAATTATTTTTGATGGCGTTCCTATTGGTTAAA<br>AAATGAGCTGATTTAACAAAAATTTAATGCGAATTTTAACAAAATATTAACGTTTACAATTTAAATATTTGCTTATACAAT<br>CTTCCTGTTTTTGGGGCTTTTCTGATTATCAACCGGGGTACATATGATTGACATGCTAGTTTTACGATTACCGTTCATCGA<br>TTCTCTTGTTTGCTCCAGACTCTCAGGCAATGACCTGATAGCCTTTGTAGATCTCTCAAAAATAGCTACCCTCTCCGGCAT<br>TAATTTATCAGCTAGAACGGTTGAATATCATATTGATGGTGATTTGACTGTCTCCGGCCTTTCTCACCCTTTTGAATCTTT<br>ACCTACACATTACTCAGGCATTGCATTTAAAATATATGAGGGTTCTAAAAATTTTTATCCTTGCGTTGAAATAAAGGCTTC<br>TCCCGCAAAAGTATTACAGGGTCATAATGTTTTTGGTACAACCGATTTAGCTTTATGCTCTGAGGCTTTATTGCTTAATTT<br>TGCTAATTCTTTGCCTTGCCTGTATGATTTATTGGATGTT
**#3**	GAGCGCAACGCAATTAATGTGCGCCCTGTAGCGGCGCATTAAGCGCGGCGGGTGTGGTGGTTACGCGCAGCGTGACCGCTAC<br>ACTTGCCAGCGCCCTAGCGCCCGCTCCTTTCGCTTTCTTCCCTTCCTTTCTCGCCACGTTCGCCGGCTTTCCCCGTCAAGCT<br>CTAAATCGGGGGCTCCCTTTAGGGTTCCGATTTAGTGCTTTACGGCACCTCGACCCCAAAAAACTTGATTAGGGTGATGGTT<br>CACGTAGTGGGCCATCGCCCTGATAGACGGTTTTTCGCCCTTTGACGTTGGAGTCCACGTTCTTTAATAGTGGACTCTTGTT<br>CCAAACTGGAACAACACTCAACCCTATCTCGGTCTATTCTTTTGATTTATAAGGGATTTTGCCGATTTCGGCCTATTGGTTA<br>AAAAATGAGCTGATTTAACAAAAATTTAACGCGAATTACAACCGGGGTACATATGATTGGGGTCTGACGCTCAGTGGAACGA<br>AAACTCACGTTAAGGGATTTTGGTCATGAGATTATCAAAAAGGATCTTCACCTAGATCCTTTTAAATTAAAAATGAAGTTTT<br>AAATCAATCTAAAGTATATATGAGTAAACTTGGTCTGACAGTTACCAATGCTTAATCAGTGAGGCACCTATCTCAGCGATCT<br>GTCTATTTCGTTCATCCATAGTTGCCTGACTCCCCGTCGTGTAGATAACTACGATACGGGAGGGCTTACCATCTGGCCCCAG<br>TGCTGCAATGATACCGCGAGACCCACGCTCACCGGCTCCAGATTTATCAGCAATAAACCAGCCAGCCGGAAGGGCCGAGCGC<br>AtAAGTGGTCCTGCAACTTTATCCGCCTCCATCCAGTCTATTAATTGTTGCCGGGAAGCTAGAGTAAGTAGTTCGCCAGTTA

	ATAGTTTGCGCAACGTTGTTGCCATTGCTACAGGCATCGTGGTGTCACGCTCGTCGTTTGGTATGGCTTCATTCAGCTCCGG<br>TTCCCAACGATCAAGGCGAGTTACATGATCCCCCATGTTGTGCAAAAAAGCGGTTAGCTCCTTCGGTCCTCCGATCGTTGTC<br>AGAAGTAAGTTGGCCGCAGTGTTATCACTCATGGTTATGGCAGCACTGCATAATTCTCTTACTGTCATGCCATCCGTAAGAT<br>GCTTTTCTGTGACTGGTGAGTACTCAACCAAGTCATTCTGAGAATAGTGTATGCGGCGACCGAGTTGCTCTTGCCCGGCGTC<br>AATACGGGATAATACCGCGCCACATAGCAGAACTTTAAAAGTGCTCATCATTGGAAAACGTTCTTCGGGGCGAAAACTCTCA<br>AGGATCTTACCGCTGTTGAGATCCAGTTCGATGTAACCCACTCGTGCACCCAACTGATCTTCAGCATCTTTTACTTTCACCA<br>GCGTTTCTGGGTGAGCAAAAACAGGAAGGCAAAATGCCGCAAAAAAGGGAATAAGGGCGACACGGAAATGTTGAATACTCAT<br>ACTCTTCCTTTTTCAATATTATTGAAGCATTTATCAGGGTTATTGTCTCATGAGCGGATACATATTTGAATGTATTTAGAAA<br>AATAAACAAATAGGGGTTCCGCGCACATTTCCCCGAAAAGTGCCACCTGACGTCTAAGAAACCATTATTATCATGACATTAA<br>CCTATAAAAATAGGCGTATCACGAGGCCCTTTCGTCGAATTCGTCGTCGTCCCCTCAAACTCTTGGGTGGAGAGGCTATTCG<br>TTTAAGGTCACATCGCATGTAATTTACTTATTCTCTGTTGTTGAGCCACCCGGGCGCCAGATTTTGTTTAAAGCTTTGTCTC<br>TTAGTTTGTATAGACAGATTCAGAGTGCAAGGTTTCGTTCGCTCGTACCTGGTTTTCCCTGGTTCTTCACAGATAGGATTTG<br>ACTTTCTACAACACTTATGCGGCTTCCTACCCGTTTGAAGGCCGATACAGGTGCTGCGCAAAATGCGGGCGAACATAGAGTA<br>TCAAAACAACGCCTTCTAATCTAGGAATATAGGGAAGATACGTATTTGCTACCATGCTTTCTTGGGTCATTAACGACCAACC<br>TCTTTTCTTTTAAAGTAGGATTGCACAATGAATGAATACACGTGGTCCGATAACTGACCAAGTAACATGGTTATCACTaGAT<br>GTCCGCCAGACGTGTGCAAACCAACCCGGGAGTTACGTCACTAATCCTTCGCTACGTCGTGAAGATATTTACTTGTGAATAT<br>CGAGGGTAATAAGATAATAGACTGTGACTAGTATTGCCAGACTGTCGCTACCTGCAACACATAACTATCCTGAGGTTACTGC<br>ATAGTACTGATTACACCCGAGTCAAAATTTCTAACTTCTAACATGTACCTAGTAACCAGCTCAATAATTATGTCAGAATATA<br>GCTCTGGGAACCCTCGGACAATTATGATACACGGTATTAATATCTTGCTTGCGTTAGCCACTTCTCATCTTTGGATACCGAT<br>TCTATTTTGCATAGCAGTTCCTTTTACACATATAAGAATTTCGCCATAGGTATGCTGCAG

Table S2. Staple sequence for the DX-based asymmetric octahedron with 63-bp edge length, for the phPB84 scaffold (2,520-nt).

Staple ID	Length (bp)	Staple sequences
1	46	TGGCGGACATTTGTGCAATCCTATTTTTTAAAAGAAAAGAGGCAAA
2	48	CGTGTATTCATTCACTAGTGATAACCATGTTACTTGGTCAGATGCTGA
3	36	AGATCAGTGCTGGTGAAAGTAAAAGTTATCGGACCA
4	47	AGAAACTGGGTGCACGTTTGGTTACGCGCGGTATTATTTCGTATTGA
5	35	AAAGGAAGAGTGCCTTCCTGTTTTTTTCTCACCC
6	48	TTTTTGCGGCATTTTATGAGTATTCAACATTTCCGTGTCGACCCAAGA
7	36	GTTTGAGGAACGAATAGCCTCTCCCCCTTATTCCCT
8	47	ACCTTAGGACGACGACTTTTCGACGTCATTTTTTAATTTATAGGCCG
9	35	ATACAAACTAATAAGTAAATTACTTTTTCGATGTG
10	48	CTCAACAACAGAGAAGAGACAAAGCTTTAAACAAAATCTGCCAAGAAA
11	36	GCATGGTAGTTGGTCGTTAATGACGCGCCCGGGTGG
12	57	AGTTTGGAACAAGTCCACTATTAAAGAACGTCGTAAAGCACTAAATCTTCCTAAAGG
13	42	GGTCGAGGTGCGGACTCCAACGTCAAAGGGCGAGTTTTTTGG
14	48	CATCACCCTAATCAAAAAACCGTCTATCAGGGCGATGGCCTAATTTAA
15	36	AAGGATCTTTTAAAACTTCATTTTCACTACGTGAAC
16	42	CTTTAGATTGAAGGTGAAGATCCTTTTTGATACTCATATATA
17	53	AGTTTAATCTCATGTTTTAAATCCCTTAAAATGTGCGCTTTTTACCCCTATTT
18	57	TCACCAGTCACAGTAGCATCTTACGGATGGCCTGATTAAGCATTGGTTTTCAGACCA
19	42	TAGGTGCCTCAATGACAGTAAGAGAATTATGCATCGCTGAGA
20	42	GAAATAGACAGAGTGCTGCCATAACCATGAGTATGGATGAAC

21	42	GTCAGGCAACTGATAACACTGCGGCCAACTTAACGACGGGGA
22	42	TAGTTATCTACCTTCTGACAACGATCGGAGGATCCCGTATCG
23	53	AAGCCCCCGAAGGATTTTACCGCTTTTTGAGCGTGACATTTTTCGATGCCTGT
24	57	GCTTCCCGGCAACTTAATAGACTGGATGGAGGTATCATTGCAGCACTTTCAGATGGT
25	42	TGGGTCTCGCGGCGGATAAAGTTGCAGGACCACCGGTGAGCG
26	48	TGATAAATCTGGAGCTTATGCGCTCGGCCCTTCCGGCTGGGCGAGAAA
27	36	GGAAGGGAAAAGCCGGCGAACGTGCTGGTTTATTGC
28	42	GCTTGACGGGGAGAAAGCGAAAGGAGCGGGCGCCGATTTAGA
29	53	GAGCCCCTAGGGCGTTTTCAAGTGTAGCGTAGGAAGCCTTTTTTAAGTGTTGT
30	32	CGCCGGGCCCTGATAAATGCTTCAATATTGAA
31	42	TGAGACAATAACAAGAGCAACTCGGTCGCCGCTATCCGCTCA
32	42	ATTCAAATATGATACACTATTCTCAGAATGACTTCTAAATAC
33	20	GTTTATTTTTGGTTGAGTAC
34	42	ACTTTTCGGGGACGTGAGTTTTCGTTCCACTGGTCAGGTGGC
35	42	GTTTCTTAGACAGCGTCAGACCCCAATCATATGATAATAATG
36	42	GTTAATGTCATGTACCCCGGTTGTAATTCGCGATTTTTATAG
37	42	GTGATACGCCTTTAAATTTTTGTTAAATCAGCAAAGGGCCTC
38	32	AAATCGGAACGAAACCTTGCACTCATCTGTCT
39	42	AGGTACGAGCGCAAAATCCCTTATAAATCAAAAGGGAAAACC
40	42	TGTGAAGAACCAGAATAGACCGAGATAGGGTTAAATCCTATC
41	20	AGAAAGTCGAGTGTTGTTCC
42	42	CCTTCAAACGGGGTCACGCTGCGCGTAACCACCCTGTATCGG
43	42	TTTGCGCAGCACACACCCGCCGCGCTTAATGCTCGCCCGCAT
44	42	ATACTCTATGTGCCGCTACAGGGCGCACATTACGTTGTTTTG
45	42	TAGAAGGATTGCGTTGCGCTCGTCTGGCCTATATTCCTAGAT
46	37	TACGTATTTTCTTCCAATACTAGTCATTTTCTATTAT
47	32	CTTATTAGTAACTCCCGGGTTGGTCACACGTC
48	48	GAAGGATTAGTGACCCCTCGATATTCACAAGTAAATATCTACTATTAA
49	36	CTGGCGAACAACAACGTTGCGCAATCACGACGTAGC
50	20	AGCAATGGCTACTTACTCTA
50	20	AGCAATGGCTACTTACTCTA
51	42	TACCAAACGACTGCACAACATGGGGGATCATGAATGAAGCCA
52	59	CTTGAGAGTTTTCGCCCTTGGGAACCGGAGCTGTAACTCGCCTTGATCGCGAAGAACGT
53	25	TTTCCAATGATGAGCAGGTAAGATC
54	42	ATCTCAACAGCCTTTTAAAGTTCTGCTATGTGATCGAACTGG

Table S3. Design parameters for pentagonal DNA origami objects.

Edge length	Scaffold			Staples		
	Required length	# of double crossovers	# of unpaired nucleotides	# of staples	# of double crossovers	# of unpaired nucleotides
**42-bp**	1,648-nt (#1)	5	133	38	52	60
**84-bp**	2,908-nt (#2)	5	133	68	110	60

**126-bp**	4,168-nt (#2)	5	133	96	170	60
**168-bp**	5,428-nt (#2)	5	133	125	230	60
**210-bp**	6,688-nt (#2)	5	133	157	290	60

# indicates the type of scaffolds in **Table S2**.

Table S4. Staple sequence for the pentagonal DNA origami of 42-bp edge-length. The sequence is represented by colors; unpaired nucleotides with blue, crossovers with orange, and the 14-nt seed dsDNA domain with green.

Staple ID	Length (bp)	Staple sequences
1	58	CCCTAATCGATGGCCTGCCATAACCATGAAGAACGTACATTCAAATATGTATTCATGA
2	56	TACTCTTTTCCCGGCAACAATTTGCGCAAATAACTGGCGATTTTAAATTTTAAATC
3	55	TTTTCGCAGTAAGAGAATTAGCACTTTCTATTTGTTTATTTGAAATGTACCATCA
4	53	ACTTTTCGGGTTCTAAATTTTCCAATGATGATGCAGTGCCACTACGTCGTGAG
5	50	GGTTGTATCTAGGTTTTACTCATATATAGTAGTTACATACACTAACTACT
6	46	CATGATTCCACTTGTCAGACCCCAATAATCTCATTTTGACCAAAAT
7	46	GGTTGAGTTAGAGCCGCCGCTACAGGGCCGTGTCGAACGCTGGTGA
8	45	ATTTCGCACATTTCGTTGCGCTCTTTATAGGTAATATTAGTGGGT
9	45	GTATCCTTTAGTTTTAAAACTTGGCGCTAGGATTGCAGCTGGATG
10	43	GGATCCCAGTTTTAAGAGTCCACTACCCTAAAGGTTTGAGCCC
11	43	CGAGCAAAATCTTAAATCAAAAGACGGCGAACGTTTTGGCGAG
12	43	AGCTCCGCATTTTTATTTATTTAAAAGGAATTCGCGCTCAGAA
13	43	CTTACGGGCGATTGTCTATCAGGGCAAGTTTTTTTTTGGGGTC
14	43	CTTTTTGATCATATACTCACCAGTCACAACGCCGGCAACTATG
15	43	AACTGTCAAGTCAGGGCAAGAGCAACTCGGTTGAGTGTACCCC
16	42	GGAGCTGGGTGCCTTCCTGTTTTTGCTCGATCGTTGAGATAG
17	42	GAGGCGGCCTGTAGAACCAATAGGCCGAAAGGGAACAAGTGT
18	42	CGGTATCGCGCTGGGAAAGCGAAAGGAGATTTTTTCAATGGC
19	42	TGACTTGGTCGCCGTCTACACGACGGGGGACCAAGGAAGATC
20	42	GCTTCAATTAATGTAGCACTAAATCGGAATTAAAGCGAAGGA
21	42	TACATCGGGAGGACAACGTGGACTCCAAGCCGTAACATGATA
22	42	TCCCTTTCTTAATGTTGACGGGGAAAGCATAGACCGGGAACC
23	41	TTCTGCGCTCTTTTTCCGGCTGGCTAATGAAGCCTAAACGA
24	41	TATGTGGCGCTTTTATCCCGTATTGGAAAAGCATTGGATGG
25	41	GTAAGATCCTTTTGTTTTCGCCCCGAGTGATAACTGGCCAA
26	41	AAGTAAAAGATTTAAGATCAGTTGGGCTTTTTTGTCATGGG
27	41	CCCTTAAGAGCGCGGAACAGGTGCCTCACTTAAGCATTGGT
28	40	ACCCGCCGCGTTTGCGGCATTTTTTATTGCTGATCTGGAG
29	40	GAGGTCGTCAAATTCTGACAACGATCAACTGGAGATAAAT
30	40	CCGATTGTTGTTATGTAACTCGCCTTACCCAGACCCTTAT
31	40	AAAGGAATCGGCGTGACACCACGATGATAAAGTGGTCTCG
32	38	GATGAACGTGACAGATCGCTGAGATCCTAAAGTTCTGC
33	37	AACAACGTAATAGACACTGGGGCCAGATGGTCCTCCC
34	37	GCTAACCGTGCACGGAAAAAGGAAGAGTATTTTCAAC
35	25	AGCGGTCGAGCGTGTGCAGGACCAC
36	25	ATAATGGTAACCCTTCTCAACAGCG
37	21	CCGGTACGCTGTTACCACCAC
38	21	GACAATTTCTTTTCAGGTGGC

Table S5. Staple sequence for the pentagonal DNA origami of 84-bp edge-length. The sequence is represented by colors; unpaired nucleotides with blue, crossovers with orange, and the 14-nt seed dsDNA domain with green.

Staple ID	Length (bp)	Staple sequences
1	58	CATCTTTCGATAGCACGAGGGTAGCAACGGTGAGGCTTTGAGTAGCTCTTTTTTAAAT
2	58	ATTAAAGTCCAGACCTAAACAACTTTCAACTCAGCGGAGTGAACGCAGTTAGCAAACG
3	55	CTTTTGAAGGACAGAACCGCCACCCTCACTTGAGCTAATGCCACTACGATGTATC
4	50	ATCGCCAGAGGTCATAAATACGCATTTGGGAATTAGCCCTCAGATGAACG
5	50	TCATATTCTGTATGGATACATAAGGCTTTTGCAAAACTAATGCAATCCTC
6	48	GCGCCAAAGAGATTGGCATTACGAGGCATAGAGACGACAACGCAAAGA
7	46	GCCGCCAGACGTTCATAAGGGAACCCGCGACCTGTTTCTCCATGTT
8	46	TTGCCCTAAAAGATTGCGAACGAGTAGATGAGGCTGAGAGGGTTGA
9	46	GTGTCGAAATCGAACTGCACCCTCAGAGCCGGTGAATTAACCTAAA
10	45	GTTTATCTACTTTTAGTAGCATTCAGAAGGAAAAAAGGACCGTAA
11	45	TAGAAAATACGATTTTGGTTAGTAAATGAATACAAATAAGATACA
12	45	GTCGCTTTAGTTTATTAGATACATTTCGCAAAACAACCCTCAGGA
13	45	ATGCAACTAACATCGGAAGCACCGTAATCAGTTATTAGTTCATTA
14	43	CGTATTTATACTTAGGACGTTGGGTCCCCCTCAATTTATGCTT
15	43	TCTGAAGATTTTTACCACATTCAAGAAGTTTTGCTTTCAGAGG
16	43	ACCAGATCAAGTTCTTGACAAGAAACCAGACCGGTTTAAGCAA
17	43	GGGGTAACACCTTAGTAGTAAATTGAAGCAAAGCTTTGGATTG
18	42	CACTGAGTAATAAGATCTACGTTAATAATCGTCATGAAACGC
19	42	TCAACCGGGAGGTTAACACTATCCGGAACGAGGCGCGCCAGC
20	42	CTCCAAAAACCGAGAAATATTCATTGAAAAGAAAATTTTAAC
21	42	GGTTTAGTTAAGAGAGATGGTTTAATTTTTACCCTATAACCT
22	42	CCACCCTCCCTGCCGCGATTTTAAGAACAGTTCAGAAGGTGG
23	42	ATTGACAATTGAGGTCATCTTTGATAAGTTTATTTGCGACAT
24	42	AGTTAAACCAATTCTAAGAGGAAGCCCGTCAGTGAGCGCGTT
25	42	AGTTTGCGCATTTTACAAAGCTGCTCATAAAGACTATAACAG
26	42	TCGCGTTCAACGTACGGTCATAGCCCCCTAGCGACGCAGCGA
27	42	CCCAAATTTAATTCTGTCTGGAAGTTTCACCCTCAAGAATCA
28	42	AATAATATTCACGTACCAGTACAAACTAGATGATATAACGGA
29	42	AAGACAGAGTACGGGAGCTTCAAAGCGACCGGATACGTTTGC
30	42	GTTTAGCGATAGTTACCCTCAGAACCGCTTATTCTAATCATT
31	42	TTGATTCGGCCGCTGTCAGACTGATAAGTGCCGTCTGCAGGG
32	42	GTGAATTCATAAATTCATTTGGGGCGCGTTTCTTAAGAACCG
33	42	CCAGTAGCCGCCTCCGCATAGGCTGGCTAACAGGTTGAATAT
34	42	TCATGAGTAATTGCCAGGATTAGAGAGTGACCAGGCCTCAGA
35	42	GTGTACAACCTTTAGATGGCTTAGAGCTGAAGTTTAAAATCA
36	42	GCCGCCAAGCCAGCCCATTAAACGGGTATTTTGCGATTGCTC
37	42	CAATGACATGGTCAGACTATTATAGTCAGGGCTTGGCTGAGA
38	42	AGGTGAAAGCTGAAAAAACGAGAATGACACCTTATTATTTCG
39	42	GTCAGACCAAAAGGTGTCACAATCAATATAAAAGAGATAAAA
40	42	GAACCTACACCCTCAACAGCTTGATACCTATATTTCAAAAAT
41	42	TTCATCGCTTTAGCTTTGCGGGATCGTCATTCCATTCAAATA
42	42	CAGGTCTCAACTTTGAAACATGAAAGTATACCGCCGCGCCGA
43	42	TTCCACATAAGCGTGGTAGAAAGATTCAATAGTAATAAGACT
44	42	AACTAAAGCATGATAATGTTTAGACTGGTATTACACATACAT
45	42	ACAACATATAGCGTACCCAAAAGAACTGGGAATTGTGTAGCA
46	42	GGCTTTTCAACGCCCGAATAATAATTTTACGGAATCCAATAC
47	42	TGCGGAAAACGAACCAGGAGTGTACTGGTTTCGTCTGAAAAT
48	41	AATATTGACGTTTTATTCATTAAAGCCACCAGAATACCAGA
49	41	CGTAACGATCTTTTTTTGTCGTCTTCCAGAATGGTGCAGTC
50	41	TTTCAGGGATTTTGCCCAATAGGAAGTGCCTTGATAGTGCC
51	41	TATAAGTATATTTGAATAGGTGTATAGAGAAGGATATTAGC

52	41	GGCCGGAAACTTTCAATGAAACCATTCATAATCATACCGGA
53	41	ACTTAGCATCCCCAGCGATTATAAAAACACGAGGGAAGGTA
54	40	ACTCCGACCTTCAGCCACCACCGGAACACCATTGACTTTT
55	40	CATCAGACGAGATTTGCTCAGTACCAGGCGGTAATAAGGC
56	40	GGGTATCAGTTGATTTACCGTTCCAGGACAGCCAAGGAAC
57	40	TAAACTGGCTCAAAACAGTTAATGCCCAGAGCCGCTTTCG
58	39	TCACCGAGAGCCACACCAACTTTGAAAGTATGATAAATT
59	39	CACCACGGAACAACCCTCGTTTACCTAAGAGCGAGGCAG
60	39	ACCAAAAAAAAGGACTTGATATTCACAATTGGTTTACCA
61	38	ACGAAAGATAAGAATACACTCCAAGCTTCAAAGTACAA
62	37	GGGGTCACCCATGTCTCCAAAAGGAGCCTTTGTATCG
63	37	CTCCTCACACCGTAATCGCCCACGCATAACTTATTCG
64	34	TAACGCCTAGCGAGAAGGTGGCAACATAGAAAAT
65	25	AATGCTGGACTAAAACCATTAGCAA
66	25	CCTTATTGAATAGACTCATAGTTAG
67	25	CATCAATTCAGCTTACCACCCTCAT
68	22	CGGAGATTAGGCACCATCACCG

Table S6. Staple sequence for the pentagonal DNA origami of 126-bp edge-length. The sequence is represented by colors; unpaired nucleotides with blue, crossovers with orange, and the 14-nt seed dsDNA domain with green.

Staple ID	Length (bp)	Staple sequences
1	60	TTTCGGAAATATCGTATGTTAGCAAACGAACGCAAGTTAGTAAATGAATGTGAAACATGA
2	58	AGAAACGTAAGCAGTTCAACAGTTTCAGCGACGATATAGCGTCCAATACTGTTCGTCA
3	58	TAGATAACAAGTACACACTGAGTTTCGTAGTATAGTGATACAGGAGTGTACTATAAGT
4	58	AAACAGCAAACGAAAGTAGCACCATTACCATAAGGCCGGAAAAAAATCTTTCTGAGAG
5	55	AATCAAATAGTTAGCGTAACAGGATTACGTATAAACAGTTACTTCAAATTAAGAC
6	55	CGAAATAACAAATAAATCCTCATGTTTGTAATTCTGTCCAGCGCCCAAACCCTCA
7	55	GGGCGACGATAACCAGCCTTTAATTGTAATCAGTTACCGACTTGAGCCATTATAT
8	55	AAAAGAATTTCTGTAAGGAAACCGAGGATAGAAAACCCGAAAGACTTCAACCTAT
9	50	AACTATTCAACCGATCACCGTCGAGATTTAGGAATACAAAAGGCACAAGA
10	50	ATTACCGACGACGAATGCTGTAGCTCAACATTAAATCAGAACCGTTGTGT
11	50	TATTCTTTTGCCAGAGAGGAAGTACATACATAAAGGTTACCAGATGGGAT
12	50	ATTCGAGATGCCCCGACTCCTCAAGAGAGATCTAAAACTGGCATGAAACC
13	46	ATCCGGTAGAATATGTGTCTGGAAGTTTGGTCAGATTAGCCGGAAC
14	46	GCAATAGTTATTTTAAAAATCAGGTCTTATTCATTTAGTAAGAGCA
15	46	CTGTCTTATCCCATCTCCAACAGGTCAGCGGGGTCGTACCAGGCGG
16	45	ATCAATTCTACTTTAGTAGCATTGAATAACCTAGCCCCTGACGAG
17	45	CCTTGATAAGTACGAAAGTACCGACAAAGATATAGTGTATCATCG
18	45	AAGTATTAAGTCCAGACTAATAACGGAATACTACGCAGCGTTTTA
19	45	TGCTCCTTTTCTTTTGACCCGGAATAGGTGTTACCGTACGCACTC
20	45	GAAGCAAAGCAGACTGGAAAAACCAAAATAGAACAACTATAGCCG
21	45	ACTACCTTTTAATCACCCTAACGGAACAACAGAGGTGAAAAGTCA
22	45	AGGCACATTCCTTCAGTTGATTCCCAATTCTCACCAGATGACCAA
23	44	TGATGCAAAATTGAGCATCAGCTTGCTTTCTTATTACATTAGAG
24	43	TTTGACTAATTTTTTACAAAATAAAGAAAAAGCCTTTTGTTTA
25	43	GACAAGACGGGTTTAACTGAACACGCGAGAAAACTTTTTTTTC
26	43	GCGCGGAACGCTTTTTTAGCGAACCATTTTCGAGTTTCCAGTA
27	43	CCTGTCATTCCTTGGGTATTAAACGTCCTGAACATTTAGAAAA
28	43	ACAACACCGAATTTTTTAAGAAAAGCAAAGACACCTTTACGGAA
29	42	CGATTTTATAAGGCAATTTTCCCTTAGATAGCGTCGGTTTAA
30	42	TTTTGCGTTGAGATAGACTGTAGCGCGTATTAATTGTTAAAT

31	42	AAGAATAATCCAAACACCCTCAGCAGCGAGAACGAGGCATTT
32	42	AAACACCAAAGACATATTATTTATCCCAAACACCGTGTAAAT
33	42	TCGGTCATTGCTTCGAATCATAATTACTACAGCCAGCATCGG
34	42	ACGTTGAACATAACAACAGTTCAGAAATTATTCATCAACTAA
35	42	GAACCGCAATGGTCGGGCTTAATTGAGAGTTGCTAAAGGCAC
36	42	AAGATTAATCGCCAAGATACATTTCGCACACCCTCGGCGCAT
37	42	GCAACTAATTCACACCGCGACCTGCTCCGGAGATTAAGGCTT
38	42	AATTGCTCGCAGTCCCACCCTCAGAACCCACCCTCAGGAATC
39	42	GAACCACGCGAACGAATTTAGGCAGAGGCTCCCGAAACACTC
40	42	AGACCGGCAGTGCCGGATTAGCGGGGTTAGCCCTCTAATCGG
41	42	AATGACCTGCTTTAGCCAAAAGGAATTAATAATAATGAAATA
42	42	CAACCTACAGACCAAGAGCCACCACCCTGACCATTTATTTAA
43	42	AGCGATACGACAGAATTGTGAATTACCTTCGGTCGGCCTTTA
44	42	CGTCGCTTTTCATCGTAGTAAATTGGGCGGATCGTTAAGAAA
45	42	CAACGCCTGAAGCCAGAGGCAAAAGAATAGAGGACGCCACCA
46	42	TGTTTAGAGCCGCCTCATCAAGAGTAATAACGGGTTATCCTG
47	42	TTAGTTTCAGAGCCAGATGAACGGTGTAAAACGAATTAAATC
48	42	AGGCTGGCACTACGTTTTGCACCCAGCTAACAGTAAATAACC
49	42	GAGCCACGCCACCCGCCAGAATGGAAAGGAATATACAATAAA
50	42	CAACATGTCATCGTATTTTCAGGGATAGAGTACCGTCTGAAT
51	42	GAGGTTTCAAGCCCAGCCGTTTTTATTTTTCAGCTAGAGCTT
52	42	TTACCGTATGGCTTAATGCAGAACGCGCACAAGCAAATAGGA
53	42	ATCGAGACTGTTTAGGTCATTTTTGCGGTCCAGTATACTCAG
54	42	ACCCATGATCACCGAGCGTCATACATGGGATAAGATCAACAA
55	42	AACGCTCACAATTTAAAATACGTAATGCCTGACCTACCCTCA
56	42	TGAGTAAAAGCAAACCTAATTTACGAGCATGTAGAGCGAACC
57	42	TCCTTATCCAAAAGAGTTTTGTCGTCTTAGGCTGACTGCCTA
58	42	AACAAAGTGGCAACATCAAAAAGATTAAGGGGGTACTTTTGC
59	42	TTTGCTACGAGAGGATAGTAAAATGTTTGGATTGCATATAAA
60	42	GATATTCTTCATGATAACGAGCGTCTTTATATGCGAAAGGTG
61	42	CCTCAAAATAAATCGTCACAATCAATAGGAAACAATTTTTTC
62	42	TGCAGATAAATCTCAATAATAAGAGCAAAAAATTCAAACGAG
63	42	GAGCCACGAGCTGATTATACAAATTCTTCCAACGCGGAAGTT
64	42	CTTTGAAACACTAACTTGCGGGAGGTTTAACATGTAGTAGAT
65	42	AAGGCTCCCACATTTAAAGGTGAATTATTGAGGGAGACAAAA
66	42	AATCTTAACCAGTATTCATTTGGGGCGCCACCGGAAGAACCG
67	42	GAGGGTATCCAATCCGGCTTAGGTTGGGTTTGGGAAGGTAGA
68	42	CCAGCAATAACCTCGCAAGACAAAGAACCCTGAACATTTCTT
69	42	TCCATTACTTGACAACCGCCTCCCTCAGCTATATTTAAAGCC
70	42	AACTGGCGCCCACGTAAAAACAGGGAAGTATTTTAATAGTGA
71	42	CCATCGAAGAGTCAGTTAATTTCATCTTAATAACACATAACC
72	42	CAGAGAGCTGACCTTTAAGACGCTGAGATAGCAGCTTTTAAG
73	42	GATATATTATGCGAACCGTAATCAGTAGGCTTAGAAAATTTA
74	42	ATGGTTTAATAGCACTGAGGCTTGCAGGTTTAATCATCAAGT
75	42	TTTCAACGAGTTAAACGTCAAAAATGAAGAAATACAAAACAT
76	42	TTGCCTTATCCTTGCGACCGTGTGATAATTGTTTAAGGCCGC
77	41	ATAAGTGCCGTTTAGGGTTGATATACACCAGTACTACAACG
78	41	ACACTATCATTTTTCGTTTACCAGACGGAGTGAGTAAAGGA
79	41	CGTTGGGAAGTTTTCTACGTTAATATTGATACCGTTGCGCC
80	41	ACAAAGCTGCTTTCAGTGAATAAGGGTAGCAACGTAGAGGC
81	41	GAGGCGCAGATTTAATCATAAGGGAACCCCCAGCTTACCAA
82	40	ATAAGATTCTAAAAACAAAGTACAACATGTTACCGATTGG
83	40	ATAATTCCTTATAGCATTCCACAGACTTGCTCAAGTGCCT
84	40	TAAGTCTATCTTTAAAGGAATTGCGACGAGGCAGAATCCC
85	40	AATATTGACGGAATATGGTTTACCAGTAAGCCCCAAAAAA
86	40	AAGATTCTCGGTTTGCTAATATCAGAGAATATGTAAATGC
87	40	AAATACGCATTATGACAACAACCATCTCATTATAATGAAA
88	40	GTATCCCAGAGCGGACTAAAGACTTTATTACCCGGAACCA
89	39	CCTGATAAACCTAGCAAGCAAATCAAGGTAAATAAATAT

90	37	AACGAGGCTTGCCCCTTATTAGCGTTTGCCTTTCATA
91	37	ATCTTTGACCGAACGCCGCCGCCAGCATTGTAGGTTG
92	25	ATTTATCCGTCACCACCAGTCAGGA
93	25	GCATCAAATCACCAAATCAACGTA
94	23	ATTGAGTCGCCAAAGGGAAGGTA
95	21	TTTAAGATTAGTTCCTTTAAT
96	21	TAAATTACCCTTTTATAGTCA

Table S7. Staple sequence for the pentagonal DNA origami of 168-bp edge-length. The sequence is represented by colors; unpaired nucleotides with blue, crossovers with orange, and the 14-nt seed dsDNA domain with green.

Staple ID	Length (bp)	Staple sequences
1	60	TCTAAAAACCATTATAAGAACTGGCTCAAATTGGGCCCATCCTAATTTAATCACTAACAA
2	58	AGAGACTATTTAACTTGCCATCTTTTCATATAATCACCGGAAGACAGGTTAGGCAGGT
3	58	GGTATTAAACAAAGTGGCAACATATAAAAGTCAAAGACACCAATGTTTTTTGCAACTA
4	58	AGAATAAGCGTAGAACCTGAAAGCGTAAGATTGGCACAGACATCACTTTTGTAGAAGA
5	55	CTGAAAGAATACCAAGTTACCGAGAAATCGTCACCCTCAGCCCCTGCCGAGCCAC
6	55	GTAACGGTTTATCAACAATAATTTGAGAACAGTTGAAAGGAATGGTCAGTGAATT
7	55	CATATTCGATAGCAGCACCGAATCAGGAAATATTCATTGAAACACTCAATTTTCC
8	55	TTTAAACGAGGCAAAAACATAGCGATAGGTGAATACTTTAGCGTCAGACATGGAA
9	50	TTCGCAAATTGAGGTCAATAGATAATACGATAAGTCTTTAATCAGTTAGC
10	50	CACTAAATCCCCCTGACCATAAATCAAATAATCAGGCTATTAATAACAGC
11	50	TAGGAGGATTAAGACCGAACGAAGTCAGGACGTTGGGAAACACGTAGAAA
12	50	AGCGCATTCAGAAAAAGTTTGCACCTTGCTTCTGTATCCTTGAAAGAATA
13	50	TAATGCCAGCGAAACAAGACAAAGAACGAAAATCGCGCCACCCTAATCGT
14	46	ATTTGTAATAGTAATCAAAAAGATTAAGGTAGCACCAAAAATGAAA
15	46	TTAGTAACCAGTACAAGGTGGCATCAATGAACCTCATTAAATCCTT
16	45	CGCCTGAATTTTAGTTAAAGGCCGCTTTTTATTCTTACCTACATT
17	45	CACCAAGGAAGTTAGACTTCAAATATCGCGTACTTGAGAGGGAAG
18	45	AAGTACGGTGATAAAGGTTACCAGAAGGAAATCATTCCGCTGCTC
19	45	CAGACGATTGTTAGCGTAATTTCATTTGAATAAATCATCCATTAA
20	45	CTAATAGATTATAATATCTTGAGATGGTTTAGCGATTTGATACAT
21	45	ACTCAAACTACCTTCTGTTTTCAGGTTTAACTAAGGCGACAATGA
22	45	TTGCTTCTACTTTTAGTAGCATTAGCTAAACAGCCAGCACATTAT
23	45	TGCAGAAAGACTTTTATCTGGTCAGTTGTATATTTTCCACAGACA
24	43	AGAAATTAAACTTATACCGATAGTGGATAAGTGCTTTCGTCGA
25	43	GAGAAGATATTTTCCAAATCAACGTTATTACAGGTTTTAGAAA
26	43	AGGTTAGGACTTTTTTTTCATGAGTGGTAATAAGTTTTTTTAA
27	43	CAGACTTCTGTTTATTTTGCTAAACCGTAACACTTTTGAGTTT
28	43	TTTATCTGATATTGTCGAAATCCGGAAGTTTTGCTTTCAGAGG
29	42	AGCAATATTATTTTGAGTAATCTTGACAATCAGTTTGCTGTA
30	42	TTTGTCAAATATAAGAGATTTAGGAATATCATCAACATCGTA
31	42	CTGACCTCCACATTGAGCTTAATTGCTGCAATCAAATGAAAT
32	42	GGAATCAAGAAACATAGAAAATTCATATTGGCTTACAACTAA
33	42	TGCAGATGGCGCATGCCCAATAGCAAGCTTAAGCCCAGCGCC
34	42	CAGACGGAGACGACCCGGAAGCAAACTCTGACGGACAAAGTC
35	42	CCTCCCGCCCTGAAAATTATTCATTAAAAACCAGAGATAAAA
36	42	CAAAGCGAGGCCGGAAACGATTTTTTGTACGCTAAAACGGAG
37	42	AGCCAGATGTAGCGATCAATATATGTGACTTAGATAACCTAA
38	42	CGCATTACTTAAATCTCCATGTTACTTATAGCGAGCGAGCTT
39	42	CACCCTCTCAGAACCGCAGAGGCGAATTCCAATCGGACAGCA
40	42	GCCAGCCGACCAGTTTTACATCGGGAGATGACCTAGTCGCTG

41	42	GTCACCGTTTAATTAGGCTTTTGCAAAACGACCTGCAAGATT
42	42	ATCACGCGCCCTAAGGATTATACTTCTGTTACCAGTTAATTG
43	42	GTTTTTACAGAAGATTATCAGATGATGGGAATCGCTCACGTT
44	42	AGGGATTCTGCAACAAGAAACCACCAGAGGCATTTAATAGAA
45	42	GGGCGCGCAATCAAACAAACAATTCGACACATGTTCCAGACG
46	42	AATTCTGTCCTTATCCAAAAGAACTGGCCGAGCATCAGAACG
47	42	TTGCGGAGGTTTACCAATAATAAGAGCATTACCGCAGGCTGG
48	42	TAGAGAGTGAGGGACGCTAATATCAGAGTCCGGTACTTTGAA
49	42	CAAAGCGGGTGAATGAATTAACTGAACAACTTGCGCGAGGCG
50	42	AATTGAGAAATCAGCGGTGTACAGACCAACATAACGTCATTT
51	42	ATTAGCAGATTGCAAATGTTTAGACTGGAAAGTACCGAGCGT
52	42	CCAATCCGAGCCTAACCAAGCGCGAAACATAGCGTTCAGAAG
53	42	CTTTCCAAAATAAGAAACGTCACCAATGATTATAGCCAATAC
54	42	TGCGGAACGATTATATTTGCCAGTTACAAAATATATCTTTGA
55	42	CCCCCAGTCGTCATTCTTTACCCTGACTAAACCATATTTATC
56	42	CTTAGAAAATCGTCTAGCGACAGAATCACGAGAATCAAATGC
57	42	AACGAAAAGGTCTCTGAATTTACCGTTCAGGCACCTAAGACG
58	42	GAAACAGGAGTCAATAATGCCACTACGACAGTAAGTCATTAA
59	42	CTGAGAATACATAACGTTTTCATCGGCATAAATCCCGTCATA
60	42	CATGGCTAAATACGTAGTGAATTTATCATACCTTTTCATAGC
61	42	ACGGGTATTTGATGTATTCACAAACAAATTTTCGGTTTAATG
62	42	CCCCTTAGCCTTGAATACAGGAGTGTACGAAGTTTAGGTCTG
63	42	AAAGACAATAAGAGGCCAAAAGGAATTAAGATGAAATATAGA
64	42	CCTGAGCTATGTAAGCAACGGCTACAGATCAGTGCCCGCCGC
65	42	GGAACCGACCAGAGCTTGAGTAACAGTGGAGGGTAATGCTGA
66	42	TCGGAACCCCGTATCCACCAGAACCACCCCTCCCTTCAATTA
67	42	TGCAAATATTCATTCAGAGCCGCCACCCAGAGCCGAAACAGT
68	42	AGAGGGTGGCGTTTTAAGGGAACCGAACCTATCATTCAGGAT
69	42	CAAATATTTGCTTTTGGATTATTTACATATGGAAAGAAACAT
70	42	GAAAGTACAGGGAGTTAATTTCATCTTCAACAATATTCACCA
71	42	AGGCTTGTTAAGAGCAGGAAAAACGCTCTGGCAGAACGGATT
72	42	GTCACACATTGCAAGCTGAGACTCCTCAATATTCGAATTTAA
73	42	CAGTAACAAATACCCACGCATAACCGATAGAGAAGTATTACC
74	42	TGGTTTGAGTACCTAATAAAAGGGACATAGAACAAGATTAGG
75	42	ATTAGCGCATCGCCGACCGTGTGATAAAGTCAGATAACAGAG
76	42	CAACAACGGGTTTTTGCTGGTAATATCCTCTGGCCGAATATA
77	42	ATAGAACTCGGCCTGCTCAGTACCAGGCTGCGCCGTTAAATA
78	42	AGAGGACCGAGGCAAATTGCTCCTTTTGAAAGGGCCCACAAG
79	42	TAGAACCTCATATGCTTGCTTTCGAGGTTTGATATGATTAGT
80	42	CTATTAGCTTCTTTAAGTATAGCCCGGATTATCAGCGTTATA
81	42	TATCGGTATAGGTGCCGTTGTAGCAATATCTTTAAGAAGGGT
82	42	CAAATTCAATAATGTGCGCGAACTGATAAAATTAATATCACC
83	42	GTACTCAGGAGCCTTATAAAGCCAACGCATTGTTTAACATCG
84	42	AATCCTGTCAACAGAAAAGGCTCCAAAAGGAGGTTTCTGTCC
85	42	CCATTAAAAAAGAGTAGTACCGCCACCCTCCAAAATAGGGCT
86	42	GAAAATCTCAGAACTGAGGCCACCGAGTAAATACCTCAATAT
87	42	TAATTGACAATTCAGAACGAACCACCAGTAATCAGCGCCACC
88	42	CTCAGAAAATTTTTCATATTTAACAACGTTCCTGATAAAACA
89	42	CATCATACCAACATGAATTGCGAATAATCCGCCACGAGAAGT
90	42	GAGGTGAGAATCCTCCTCAGAGCCACCAACTAAAGGTAATTT
91	42	AGGAACACCCTCATGGAACGGTACGCCAGGCGGTCGAATTAT
92	42	AGGCAGAAGGAGCGAGTATTAACACCGCTTAGACATTTCAGG
93	42	GATAGCAGAGTGAGTCGAGCCAGTAATACGGAACAAGTGCCA
94	42	CATTTTGAGAGAATCAACAGTTTCAGCGAGCCCAACGATTAA
95	42	CGCTGAGAGGAGGCTAGGAACCCATGTACAACTTTATAAAGT
96	42	AGGCTTAAGATAACGACATTCAACCGATTACCTTTTAGTAAG
97	42	AAACCCTAGCTGAAAAACTACAACGCCTCGTCTTTCAGCTAA
98	42	AAGTATTCGCGCCTATCTAAAGTTTTGTGTAGCATTCATTTG
99	42	TAAATATCAACAGGAACCCTCGTTTACCTCAATCATAGCGAA

100	42	ACCTTATATTTCAACCTGAACAAGAAAAAGAGCCGAAGGTTA
101	42	ACCAAAAGCCGGAAGGAGGTTTTGAAGCGACGGGATATCACC
102	42	TAATAACATAATCGGCTTGCCCTGACGAGAAGAAAGATTCCC
103	42	CCAATCAGGAATACTACGCAGTATGTTAAACAGTTAATCTAC
104	42	GTTAATAGAATAAGGCTGTCTTTCCTTACCGAGGATAGAAAA
105	42	ATTCAGTAAACGAAGTTTCATTCCATATGCAAACGAACGCAA
106	42	TACATACTCTGGAACTAACGGAACAACATAACAAAAAGAACG
107	42	AGCAACATGACCAATTCTAAGAACGCGAAATTGAGGGGAAGG
108	41	AAACATCAAGTTTAAAATTAATTACACCTTTTTATCGGCTT
109	41	TGCACGTAAATTTAATAAAGAAATTACACCGGAATATTACT
110	41	TGCCCGAACGTTTAATTTTAAAAGTAAAGGTAAATTCTGTC
111	41	TTTTTAAGAATTTAGCAGATAGCCGAACCAAGTATCTCATC
112	41	ATAGCAGCCTTTTGAGAGAATAACAATTTTGCACTTACAAT
113	40	GGGTATCATCGCCCTGAATCTTACCATTAACGTCATTACC
114	40	CGGGGGGCTTTGGGGTTATATAACTAAAAAGAACACCACC
115	40	GAGGGGAATTTCAAGCCTGTTTAGTATACCATATGAATGG
116	40	CGTCAATGAATTGACGACAATAAACAAACTCGTAAATATC
117	40	GATTCAGAACCGCAAGCAAGCCGTTTGCTATCTAGTTTAT
118	39	TTGACGCTCCATATTTCGGAACCTATGCGGGAACTTTTT
119	39	GCCCTCATATTATAACCTGTTTAGCGCAAATCGATTTAG
120	37	ACCGACATTGAGTAAGCAAATGAAAAATCTTATCACC
121	37	AGTTGCTTAAAAACCCATTTGGGAATTAGATCAAAAT
122	34	AGTAGTATTATACCGTAGATTTAGTTTGTATCTT
123	25	CAGCATTCCAGAGCGATGATGAAAC
124	25	AATAACAATATTTTTCAAAATTATT
125	25	GCTCAACCGGAATATACCGAAGCCC

Table S8. Staple sequence for the pentagonal DNA origami of 210-bp edge-length. The sequence is represented by colors; unpaired nucleotides with blue, crossovers with orange, and the 14-nt seed dsDNA domain with green.

Staple ID	Length (bp)	Staple sequences
1	58	AGGTGCCGACCTGACGAGGAAACGCAATACTGGCTGTAAGAGCAACACTATTCCCTCG
2	58	TTATTAGACGGGTAGTCCAGACGACGACAATAACATGTTCAGCCAGCTTTGCACCGCT
3	58	TATTGACTTTGAAATAAATAAGGCGTTAAATATAAACACCGGTTTCCCTTTCCTTGAA
4	55	AGAAAACAAATCGCAGCAGCACCGTAATCCCCCAGAACGCCAACATGTAGGGCCT
5	55	ACAGGTAATAGCTATGATAGCCCTAAAATCAAAGGCTTCAAATATCGCGGGTAAT
6	50	CTTCGCAATTAATTAATTTAACCGATTATACCAAGCCATCGATGCAGAGG
7	50	TGAGCGAAGATTCATCGAAAGAGCGAAAAACCGTCTCGCGAACTCTTACC
8	46	CCAGCAGAAATCGGGGTCATTTTTGCGGTTAACGTCACTGAGTTTC
9	46	AAAGGGACTAAAGGAAACAGTTCAGAAACAGACGAACTTTAATCAT
10	46	AAATTAATTAGAATCGCTATTAATTAATAATCATACGCAGACGGTC
11	45	TCTGGTGCCGTAATTCTAAATACGTAATGCCCGGTCATAATATAC
12	45	AAGCGCACCAACAGGAGTGTTGTTCCAGAAACAGAACCCTCAGAA
13	45	AACATAGCGACGTGTGAGAGGACAGATGAACGGAGGGAACTTGCC
14	45	AGATATTCTACTTTAGTAGCATTGTCTGGCCAGGAATCCGAGGTG
15	45	GAGAGATAACGCAAGAAATTAAAAATACCGATAAAGAATCGAGCT
16	45	AAAATCAGGTAGGCATACATTATACCAGTCAAGGAAACAAGCGTA
17	45	TTTGTATGGCTTTTTAATTGCTGAATATAATTATTATTCACAGAC
18	44	GCTGGCGAATGCTTTGGACAGAATCAAGTTCACTAAACAGAGGC
19	43	TCAAAGCGTAGTTCAGGTTTAACGTAAAACGACGTTTGCCAGT
20	43	CCGTTTGCCCGTTATTAATTTTAAGCTCACTGCCTTTCGCTTT
21	43	AACTGGCCAACTTAGAACCCTTCTGTAAAGCACTTTTAAATCG

22	43	GAATTAGCAATTTTTGATTAGTAAACGTATAACGTTTTGCTTT
23	43	TCGAGAAAATCTTATCACCTTGCTTCCTGTTTGATTTTGGTGG
24	42	GGCCAACTAGAAGTCTGGTAATAAGTTTAGGAATTCCCGACT
25	42	CAACTAATAACGGGTACATTTGAGGATTGCGCGGGAATAACC
26	42	AACGGAGCGGAAACCAATTACCTATCCTGAGAAGTACCATTA
27	42	TGCGGGAAATGGTCGAGAGGCGGTTTGCTAGATAAGTCAGTG
28	42	CCGTCAAGTATTGGAGATACATTTCGCAGGTTTTGAAAGGAA
29	42	CCTTGAGAGAATAGAAGCCTTAAATCAAGACCATTGCGCCAG
30	42	TATAAAATGAATAAAAATGTTTAAAATGCTGATGCAGCTGCT
31	42	TCAGTTGTGCAGCAAACTAAAGTACGGTTCTTTCCTCGTCTT
32	42	GGCTGAGAGTTTTGAGAGCCTAATTTGCAATATGCAGCGGTC
33	42	TCCTTTTGCAGCCTATAGGAACCCATGTTATAGCCCTGAGAG
34	42	GCAAACTTTAGACGCCCTCAGAGCCACCTACCGCCGGTGAGG
35	42	TCAAAGCACACCCTAATTGAGTTTCAGAACCGCCAGGAGAAT
36	42	ATTGCATACATTCATAAAACGAACTAACTTTTTAAATTAGTC
37	42	ACAGCCAGCTGTAGCCAGCAGGCGAAAAGAACCTCGGGGTTT
38	42	CCCTCAAATAGCGAGCTTGAGATGGTTTCCTTATTAGTAATA
39	42	GCGTCCAAATAGTAGGCTTGCCCTGACGTAAAGGTAAATGGA
40	42	CTCCGGCGACAAAGTTACCCAAATCAACATAAGTTAACGCTC
41	42	GAATTTAATTTCATTGACCTTCATCAAGTACCAGCAATATCC
42	42	CTTCTGTCCTGTTTTGTTACTTAGCCGGCTTGAGCATCACGC
43	42	GGAAACAAAAGCCATCATCGCCTGATAACACCATTGTTTTTA
44	42	CGCAACTAGCCAGTGAGGCAAAAGAATATGCCTTTCGGATTC
45	42	CACGCTGCAATCAAAAGAGAAGGATTAGATAGTTAAAAATAA
46	42	AGTTTGAGAACAAGAGACAGCATCGGAACCTCAGACATATCA
47	42	TCACGTTGAAACCAGTTAAAGGCCGCTTCACCCTCGTTTGGA
48	42	TCTCCGTAACGGGTCCACGCATAACCGACAGCATTCTGATTA
49	42	TTTCATCAGCCGTTCTTGATACCGATAGGATATTCAACAAAG
50	42	AGCCCTCGATTAGCAAATATCAAACCCTGTTTGCCCTCAACA
51	42	TGTTTAGCGAACCTGCGAATAATAATTTGAGTGTAATTAGAC
52	42	TTAGTTTGATTAGTGTTTCAGCGGAGTGTAACAGTATTAGAG
53	42	TTCCATATCTTACCAATGAATTTTCTGTGGAACCTAAGGTTA
54	42	TGTTTTACAGTTACGCGTAACGATCTAAACTCCTCTATCTGG
55	42	GGTGGTTCTAATAGGCCCGTATAAACAGTTCAACATGCTATT
56	42	GAAAATAGATAAGACAAAATCCCTTATATGCCACGCGGAATA
57	42	GGATAGCCACCGTAACCGCCTGCAACAGAATCAAATAATTGC
58	42	GGTGTATAAGCCCATTACAGAGAGAATAGTACCTTAGAATAG
59	42	CCCGAGATATTAACCTCAGGAGGTTTAGACCCTCAAACAGGG
60	42	CGGTCAGTAGGGTTGTCAGGATTAGAGAACATAAATTTTCAG
61	42	TCCAGACTGAAAGTAACAGTTGAAAGGAACCGCCTAGTTTCA
62	42	TAACTGAGAACCAGCAAGAGTCCACTATACGAACCGCCCAAT
63	42	AATAAGACCACAAGGAACAAAGTCAGAGTTTTAATCGTGGAC
64	42	ACGAGCGGTCTGGAGGCCCTGAGAGAGTGCAAATCATTAAGA
65	42	TTTAATGATCAGGGATTAAGAGGAAGCCCAGTTGACATTATT
66	42	GAAGCCCGGAACAAGATTTAGGAATACCCAAAAAGCGATGGC
67	42	CCACTACAATGGCTGAAAAGTAAGCAGAACGTTAAACTAATG
68	42	AAAATCTTAGCCGAAGACAATATTTTTGGTGAACCAAAGCGG
69	42	CAGATACCAGAAGCATCACCCAAATCAAGTGGCACACAAAGT
70	42	AGAATACGTTTTTTCTGACTATTATAGTATAACGCGGGAAGA
71	42	TACCAGAGGACGTTCAAAAGGAATTACGCTTTACCGGGGTCG
72	42	AACAACTTTAATGCAGGAGCACTAACAATTTCTTTAGTAGAT
73	42	AACCAAAATGCTTTGAGCCCCCGATTTACACGACCACGCAGT
74	42	GAGTAGTCAAACGTAGATTCACCAGTCAGAGCTTGTGAATCC
75	42	ATGTTAGAAATTGGGAGGCTTTTGCAAATATTCATACGGGGA
76	42	AAGCCGGCATTGGCAGAAAATACATACAAGAAACATTGCCAG
77	42	TTATTTACGAACGTGGAATCGTCATAAAAGAAGTTCCAGAAC
78	42	AGGGGGTATACTGCGGCGAGAAAGGAAGTCGTCTGGGCAACA
79	42	CATTCAGGAAACGCATTTTGACGCTCAAGGAAGAACTGGATA
80	42	GGAGCGGTACCTACAAAGACACCACGGAGTAACAAAAATCCA

81	42	ATGGAAAGCGCTAGGGGTTATATAACTATATGTGAAGCGAAA
82	42	ATCGCAATTAGGTTGGCGCTGGCAAGTGCAGGAAATATTTTG
83	42	GAACCGGCAATAGAGCCAGCCATTGCAATAGCGGTTTTTAAC
84	42	TCACAATATATTCAAACGCGAGAAAACTACTACCTCACGCTG
85	42	CGCGTAATATTACCAAATTCATATGGTTAGTAATCATATATT
86	42	AGAACAACCACCACCATAGGTCTGAGAGTTTTCAATTGACAA
87	42	TTAGTTATCAAAATACCCGCCGCGCTTATGCTGGTGCCAAAG
88	42	GCGCATAGGCGACACAAACTATCGGCCTATGCGCCCAATAGT
89	42	ACAAAAGGGCTGGCCTTCTGACCTAAATGAAGAGTGCTACAG
90	42	GGCGCGTAAGAACTTTCAACCGATTGAGGGTGTACTTTGAAA
91	42	TGAGTAGACTATGGGATTAAGACGCTGATTAATGGAGACCAG
92	42	TACCGACTAGCTTATTGCTTTGACGAGCTAACATCAGGTAAA
93	42	TTGCACCGCGAACGTCACCAGTGAGACGTATCTTTCCCCTGC
94	42	GAAAAAGAAATCGTCAGAGCGGGAGCTATCTGTCCCATTTGG
95	42	CGCGACCAGCCAGCACCGAGTAAAAGAGAACAGGAAACCTTG
96	42	GAATTAGTGCTCCAAGTATCATATGCGTAGTGAATGGCCGAT
97	42	TAAAGGGTGAGGCCAAAATCACCAGTAGATTGTGTATTCTTA
98	42	TAATCAGATTTTAGAATCAATATATGTGTATACAACGAAATC
99	42	CCAGTATGTACATAACAGGAACGGTACGCCAGAGATTTTAAT
100	42	GCAAGGCATTTGTAACGCTCAACAGTAGTTACCTTGCAAAAG
101	42	AAGATGATTCATTTGTCACCAATGAAACGCGAAACTTGAGAA
102	42	CGAATTATGAAACACAATTTCATTTGAAGGCTTAAAAAGTAC
103	42	TCTAAAAGGCAACATGATTCCCAATTCTCAGCTACTTTGCTA
104	42	GCCTGATAGGGGGAAGGGCGATCGGTGCATTTAGGACACTCA
105	42	ATTTTCGGTTGGGATGTGCTGCAAGGCGACAATAAAGCGTCA
106	42	AACCTAAGCGCGTTTTACATCGGGAGAAATTAAGTCAGGCTG
107	42	GACTGTAAACGAAAAATAAGAGAATATACGCCATTTGGGTAA
108	42	CGCCAGGGTACCTTTTCATCGGCATTTTACTACGAGACAAAA
109	42	AGTAACAGTTTTCCGGCAAAGCGCCATTAAGTACCAGGCACC
110	42	GGTAAAGGAAACCACAGTCACGACGTTGTCAGATGAGCCCCC
111	42	CTATTTCATGGGATAATTTTATCCTGAATAACAGTGCTGATT
112	42	GCTACAGAGCCACCTAAAACAGAAATAAGCTTGCAAGATCGC
113	42	GCCTGTTCTCAGGATGCCTGCAGGTCGATTGCACGACCGGAA
114	42	AAATTATCTCTAGAACGACAGTATCGGCTATCAACAGCAACG
115	42	CCGCCTCCGAGGGTAATAGATAAGTCCTGGGGACGGGATCCC
116	42	CGGGTACAACCTACGCCGCCACCCTCAGCAGCGAAAAAAATA
117	42	CCCTCAGAACCGCCAATGGAAGGGTTAGCGAGCTCATCTGCC
118	42	ATATCCCACCGTGCGAATTCGTAATCATTCTGAATACCCTCA
119	42	TTATACTGGTCATAATGGGCGCATCGTAATCCTAAATCGTCA
120	42	GAGCCACTTGCGGGTTTACGAGCATGTAGGTGTAGGCTGTTT
121	42	CCTGTGTCCTGATTAGAGCCGCCACCAGGCAGGGAATCAATA
122	42	GAGGCTTAACCACCTTCATCAATATAATGAAATTGGGATAGG
123	42	ATCGGCTCGTAATGTTATCCGCTCACAAATGGCAAACCAGAG
124	42	TCAGATGTTCCACAAACGGCGGATTGACGTCTTTCGGTCGCT
125	42	CCGCCGCTATATTCCTTATCATTCCAAGGGGAACACAACATA
126	42	CGAGCCGCATATTCGACAGGAGGTTGAGCCATCGCATTAAAC
127	42	ACAACAAGCAGGTCAGCGGAATTATCATGAAGCATGTCGGAT
128	42	CAAGTACACAACCCAAAGTGTAAAGCCTCAGAAGGAGACGAT
129	42	AAACCACGGGGTGCAATGTGAGCGAGTACGCACTCGACAATG
130	42	TGGCCTTTTGCGCCATCGAGAACAAGCAAACATTACTAATGA
131	42	GTGAGCTTTTGCGGACAAACAAATAAATTAAACAGTTTATTT
132	42	AATTTCTCCTCATTAGTAACATTATCATAACTCACAGCCAGC
133	42	TCATCGTTTCCTGTATTAATTGCGTTGCAAGTTTGAAAGCCA
134	42	GCCCTTCATTGAGGATTATTCTGAAACAGTTAGTAAACGCTA
135	42	TCTCCAAACATGGCGACAACTCGTATTACGGGAAAAAAGGTG
136	42	GGTATTCGAGCTGACCTGTCGTGCCAGCACAATTCTTTTGAT
137	42	TTTACAATGCATTATTCATTTGGGGCGCTAAGAACTTGAAAA
138	42	GATACAGTTTCACGGCGAGGCGTTTTAGCTATATTATGAATC
139	41	TGTGAATTACTTTGCGATTTTAAGAAATAACGGATCAAAAG

140	41	AATCATAAGGTTTGAACTGACCAACGGAAATTATTAAAGGT
141	41	ACTTTTTCATTTTAGTTTCCATTAACGTTTGCCATTCATAA
142	41	AGCCTTTAATTTTCGGTTTATCAGCAAGCGCAGTTAATTTA
143	41	GTCACCAGTATTTTACAACGCCTGTTACCAGGCGTGTGCCG
144	40	TTCCGCAAATGAAGGGTTGATATAAGACCGTAACAAAAAT
145	40	GAACCCATTCTGGCATGATTAAGACTAATTTCACGATAAA
146	40	CCTCGCCGTTGTATCACCGTCACCGAAACGAGGATTACTA
147	40	TCTTTGACAGTAGCAATACCAAGTTACAAATATTACGCCA
148	40	GCCAAAGAAATTATCACCGGAACCAGAGGCTTTAGAACGC
149	40	CCAGTAATCCTTCCAGTAAGCGTCATAAAAAAGCTTATCC
150	39	CCGCCACCCAAACCAGCAGAAGATATTTGGAAACCGGAA
151	39	TCCAACGCATCGCCACAATGAAATAGCAGACTAATATCA
152	37	GAATGGATTGCTTTATTACCGCGCCCAATATCAAATC
153	37	TGCTCAGAGCATTCTATCCCAATCCAAATATCGATTT
154	25	ACTCCAGCTAATGCGAGGACTAAAG
155	25	GCATCAATAGAAGGGCTCCAAAAGG
156	21	TTTACACGAGATTATAAATCA
157	21	TCGCCATCATTTAAAACATCA
